# Plastome phylogenomics provide new perspective into the phylogeny and evolution of Betulaceae (Fagales)

**DOI:** 10.1186/s12870-022-03991-1

**Published:** 2022-12-24

**Authors:** Zhen Yang, Wenxu Ma, Xiaohong Yang, Lujun Wang, Tiantian Zhao, Lisong Liang, Guixi Wang, Qinghua Ma

**Affiliations:** 1 State Key Laboratory of Tree Genetics and Breeding, Beijing, 100091 China; 2 Key Laboratory of Tree Breeding and Cultivation, National Forestry and Grassland Administration, Research Institute of Forestry, Chinese Academy of Forestry, Beijing, 100091 China; 3 National Innovation Alliance of Hazelnut Industry, Beijing, 100091 China; 4 Hazelnut Engineering and Technical Research Center of the State Forestry and Grassland Administration, Beijing, 100091 China; 5 Forest Botany and Tree Physiology, University of Goettingen, 37077 Goettingen, Germany; 6 Guizhou Academy of Forestry, Guiyang, 550005 China; 7 Anhui Academy of Forestry, Hefei, 230031 China

**Keywords:** Betulaceae, Plastome structure, Molecular dating, Biogeographic history, Character evolution

## Abstract

**Background:**

Betulaceae is a relatively small but morphologically diverse family, with many species having important economic and ecological values. Although plastome structure of Betulaceae has been reported sporadically, a comprehensive exploration for plastome evolution is still lacking. Besides, previous phylogenies had been constructed based on limited gene fragments, generating unrobust phylogenetic framework and hindering further studies on divergence ages, biogeography and character evolution. Here, 109 plastomes (sixteen newly assembled and 93 previously published) were subject to comparative genomic and phylogenomic analyses to reconstruct a robust phylogeny and trace the diversification history of Betulaceae.

**Results:**

All Betulaceae plastomes were highly conserved in genome size, gene order, and structure, although specific variations such as gene loss and IR boundary shifts were revealed. Ten divergent hotspots, including five coding regions (*P*_i_ > 0.02) and five noncoding regions (*P*_i_ > 0.035), were identified as candidate DNA barcodes for phylogenetic analysis and species delimitation. Phylogenomic analyses yielded high-resolution topology that supported reciprocal monophyly between *Betula* and *Alnus* within Betuloideae, and successive divergence of *Corylus*, *Ostryopsis*, and *Carpinus*-*Ostrya* within Coryloideae. Incomplete lineage sorting and hybridization may be responsible for the mutual paraphyly between *Ostrya* and *Carpinus*. Betulaceae ancestors originated from East Asia during the upper Cretaceous; dispersals and subsequent vicariance accompanied by historical environment changes contributed to its diversification and intercontinental disjunction. Ancestral state reconstruction indicated the acquisition of many taxonomic characters was actually the results of parallel or reversal evolution.

**Conclusions:**

Our research represents the most comprehensive taxon-sampled and plastome-level phylogenetic inference for Betulaceae to date. The results clearly document global patterns of plastome structural evolution, and established a well-supported phylogeny of Betulaceae. The robust phylogenetic framework not only provides new insights into the intergeneric relationships, but also contributes to a perspective on the diversification history and evolution of the family.

**Supplementary Information:**

The online version contains supplementary material available at 10.1186/s12870-022-03991-1.

## Background

Betulaceae (Fagales) is a relatively small but morphologically diverse family, comprising six extant genera and approximately 160 shrub or tree species [1]. Betulaceae species are mainly distributed in the northern temperate zone, and a few extend to subtropical highlands of Central and South America [2, 3]. Morphologically, the family is characterized by typical synapomorphies, such as doubly-serrate leaves, compound catkins, and bract wrapped nuts [4], while each genus derives highly specialized traits which may play key roles in lineage-specific adaptive radiation [2]. As a geographically widespread and morphologically diverse group, Betulaceae has served as a model system for exploring taxonomic, systematic, and biogeographic issues [5–7]. To understand the diversification history and evolution of key traits, however, a robust phylogeny is required.

The generic delimitation and infra-familial relationships within Betulaceae have been examined through a series of approaches, including morphological characters [8, 9], fossil evidence [8, 10, 11], and molecular analyses [2, 4, 12]. It is now well established that Betulaceae is resolved into two subfamilies: Betuloideae (*Betula* L. and *Alnus* Mill.) and Coryloideae (*Corylus* L., *Ostryopsis* Decne., *Ostrya* Scop., and *Carpinus* L.). Nevertheless, different viewpoints on the phylogenetic relationships and morphological evolution among genera have been frequently proposed, with most controversies fastening on the divergence order among genera [9, 13], paraphyletic or sister relationships between *Alnus* and *Betula* [2, 14], phylogenetic status of *Ostryopsis* and *Corylus* [8, 15], and whether *Ostrya* and *Carpinus* were reciprocal monophyly or mutually nested [2, 16]. Throughout the above studies, the controversies can be attributed to morphologically parallel or convergent evolution, incomplete taxa sampling, and limited sequence variation (e.g., single or combined ITS, *Nia*, *mat*K, and *rbc*L). Thus, detailed relationships within Betulaceae need to be explored based on extensive taxon sampling and utilizing genome-level molecular sequences.

The relatively rich fossils for Betulaceae have promoted molecular-clock studies that inferred divergence ages among or within genera [4, 8, 12]. Nevertheless, those results varied greatly with different dating strategies and datasets. Assuming a split at either 45 or 80 Ma between *Alnus* and *Betula*, Bousquet et al. estimated the substitution rates of *rbc*L gene as 0.37 or 0.67 × 10^−4^ per site per million years [8]. Based on nuclear ribosomal ITS and 5S spacer sequences, Forest et al. inferred the median ages of crown lineages of Betulaceae, Betuloideae, and Coryloideae as 119.0 Ma, 109.3 Ma, and 70.2 Ma, respectively [12]. Using both chloroplast and nuclear DNA sequences, Grimm and Renner revealed the stem groups of Betulaceae could date from the upper Cretaceous, two subfamilies from the Paleocene, the crown group of six extant genera from the middle Miocene [4]. Although previous analyses have provided some insights into evolutionary history of Betulaceae, these estimates were inferred from unrobust phylogenetic framework constructed by a small number of DNA fragments, casting doubt on the inferred ages and hindering the development of a comprehensive understanding of origin and diversification.

Due to the highly conserved structure, uniparental inheritance, and composition of large numbers of single copy genes, plastome phylogenomics has been widely used in resolving problematic relationships within angiosperms [17–19]. Comparative genomics also provide a new perspective into plastome evolution, such as structural rearrangements, gene loss, and divergence hotspots. Particularly, the contraction or expansion of IRs has significant influence on the evolutionary rate of plastome [20–22]. Those events may function as effective phylogenetic signals. In Betulaceae, although some representative plastomes of each genus have been sporadically released, most studies mainly centered on describing the plastome characteristics of single species, and/or performing comparative and phylogenetic analyses based on a small number of plastomes (one or a few plastomes per genus) [23–26]. These studies may have contributed to the development of Betulaceae plastome resources, but are not sufficient to elucidate the overall structural variation and phylogenetic discordance, especially the non-monophyly of genera. So far, a pan-plastome study has not been conducted due to the imbalance between plastome number and species number in each genus. As the accumulation of Betulaceae plastomes, it is sufficient and necessary to conduct a comprehensive pan-plastome research to better understand the phylogeny, diversification, and evolution of the family.

In this study, we verify the power of whole plastomes to resolve the phylogenetic and evolutionary questions of Betulaceae by analyzing extensive plastomes of 109 accessions. All these plastomes were newly assembled or obtained from GenBank, representing six extant genera of Betulaceae and outgroups. Our objectives are as to: (1) elucidate plastome structural evolution of Betulaceae and reconstruct phylogenetic relationships among extant genera; (2) infer the origin and diversification history of Betulaceae; (3) trace the evolution of taxonomically important morphological characters across the family.

## Results

### Characteristics of Betulaceae plastomes

The plastomes of Betulaceae species varied little in genome size, ranging from 158,647 bp (*Betula pubescens*, MG386370) to 161,667 bp (*Corylus avellana*, MN082371). The average genome length decreased successively from *Alnus* to *Ostrya*: *Alnus* (160,690 bp), *Betula* (160,529 bp), *Corylus* (160,094 bp), *Ostryopsis* (159,630 bp), *Carpinus* (159,338 bp), and *Ostrya* (159,230 bp). All plastomes exhibited the typical quadripartite structure of angiosperm plants, comprising two inverted repeat regions (IRa and IRb) (25,929–27,567 bp) separated by a small single copy region (SSC) (17,167–19,535 bp) and a large single copy region (LSC) (87,808–90,272 bp). The total GC content of these plastomes was highly similar (35.9–36.5%). In addition, a total of 121–136 genes were encoded, of which 14–23 genes were duplicated in the IR regions and 107–114 were single copy genes. Among the unique genes, 78–80 were protein-coding genes, four were rRNA genes, and 24–32 were tRNA genes. Of these protein-coding genes, 78 genes were commonly shared by all Betulaceae plastomes and two genes (*inf*A and *ycf*15) were lost in most of the plastomes (Tables 1, S1 and S2).

**Table 1:** Sampling information, accession numbers, herbarium vouchers and structural features of 16 newly sequenced genomes

Species	Sampling locality	Length	LSC	SSC	IR	Gene	GC%	Accession Numbers	Herbarium vouchers
*Ostryopsis davidiana*	Neimeng, China	159,286	88,568	18,588	26,065	129	36.5	MH628451	Os-202104-dav
*Alnus cremastogyne*	Sichuan, China	160,538	89,074	19,094	26,185	129	36.4	MH628453	Al-202104-cre
*Corylus mandshurica*	Jilin, China	159,828	88,850	18,782	26,098	128	36.4	MH628448	Cor-202104-man
*Corylus sieboldiana*	Saitama, Japan	159,870	88,865	18,795	26,105	128	36.4	MH628456	Cor-202104-sie
*Corylus wangii*	Yunnan, China	159,825	88,743	18,870	26,106	129	36.5	MH628454	Cor-202104-wan
*Corylus yunnanensis*	Yunnan, China	160,085	89,043	18,840	26,101	127	36.4	MH628461	Cor-202104-yun
*Corylus heterophylla* var*. sutchuenensis*	Anhui, China	159,887	88,886	18,805	26,098	128	36.4	MH628447	Cor-202104-sut
*Corylus jacquemontii*	Sindh, Pakistan	159,862	88,846	18,818	26,099	128	36.4	MH628458	Cor-202105-jac
*Corylus kweichowensis* var*.* *brevipes*	Jiangxi, China	159,822	88,850	18,776	26,098	128	36.4	MH628452	Cor-202105-kwe
*Corylus americana*	Michigan, USA	159,912	88,895	18,813	26,102	128	36.4	MH628446	Cor-202105-ame
*Corylus colurna*	Tbilisi, Georgia	159,842	88,873	18,773	26,098	128	36.4	MH628457	Cor-202106-col
*Corylus cornuta*	Minnesota,USA	159,857	88,877	18,796	26,092	128	36.4	MH628460	Cor-202106-cor
*Corylus cornuta* var.* californica*	California, USA	159,915	88,867	18,844	26,102	128	36.4	MH628459	Cor-202106-cal
*Corylus fargesii*	Gansu, China	159,838	88,879	18,777	26,091	127	36.4	MH628462	Cor-202107-far
*Corylus ferox*	Yunnan, China	159,825	88,742	18,869	26,107	128	36.5	MH628455	Cor-202107-fer
*Corylus ferox* var. *thibetica*	Shanxi, China	160,358	88,357	18,769	26,616	128	36.4	MH628450	Cor-202107-thi

### Comparative genomics and divergence hotspots

**Fig. 1:**
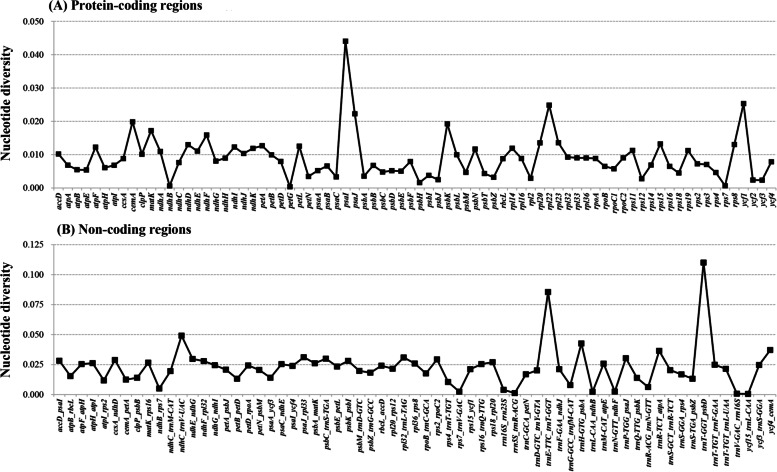
Comparison of the nucleotide diversity (*P*_i_) values across 31 Betulaceae plastomes (covering major lineages within Betulaceae). **A** Protein-coding regions. **B** Non-coding regions

The global plastome divergence of major lineages within Betulaceae was visualized using mVISTA. The plastome-wide alignment revealed globally high similarity (Fig. S1). For the entire plastomes, the SSC and LSC regions displayed marked divergence than the IR regions. The variable proportion of non-coding regions was greater than that of protein-coding regions, and the divergence hotspots were mainly located in the intergenic spacer regions (Fig. 1). For the 78 coding regions, the *P*_i_ value for each locus varied from 0.0004 (*pet*G) to 0.0440 (*psa*I), with five loci (*psa*I, *ycf*1, *rpl*22, *psa*J, and *cem*A) over 0.02 (Table S3). Among the five coding hotspots, *rpl*22 gene showed moderate variation level (*P*_i_ = 0.0249) and appropriate nucleotide length (531 bp), making it an excellent potential DNA barcode. Its amino acid alignment across all plastomes was shown in Fig. 3B. For the 65 non-coding regions, nucleotide variability ranged from 0.0005 (*ycf*15-*trn*L_CAA) to 0.1100 (*trn*T_GGT-*psb*D), with the top five noncoding hotspots (*P*_i_ > 0.035) selected as *trn*T_GGT-*psb*D, *trn*E_TTC-*trn*T_GGT, *ndh*C-*trn*V_UAC, *trn*H-GTG_*psb*A, and *ycf*4_*cem*A (Table S3). These divergence hotspots can be used as potential DNA barcodes for phylogenetic analyses and species delimitation.

### Boundaries between IR and SC regions

**Fig. 2:**
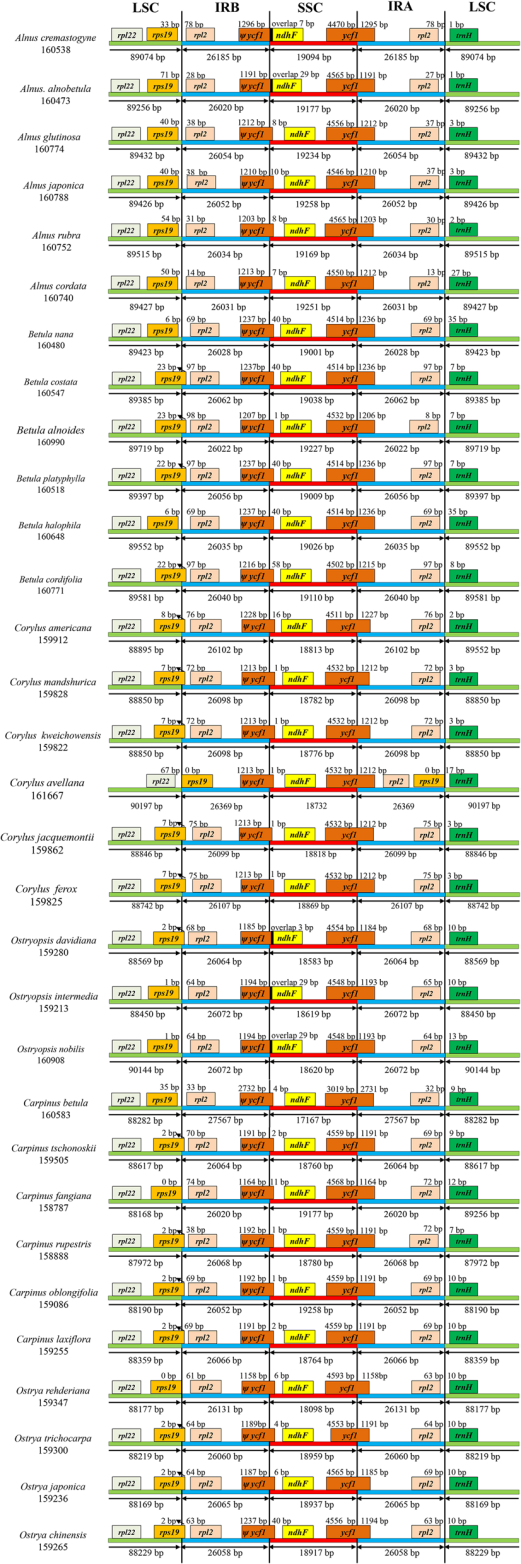
Comparison of the IR/SC junctions among 31 Betulaceae plastomes (covering major lineages within Betulaceae)

Betulaceae plastomes were relatively conserved, however, some structural variations were still identified, especially at the boundaries between IR and SC regions (Fig. 2). Junctions of the IRb/LSC region were located between the *rps*19 and *rpl*2 genes in 13 species, and within the *rps*19 gene in 17 species, with only *Corylus avellana* (MN082371) locating between *rps*19 and *rpl*22 genes. All Betulaceae plastomes had the IRb/SSC junction within the pseudogene (ψ) *ycf*1, ranging from 1 to 58 bp from the boundary. The *ndh*F gene overlapped 7 nucleotides with the ψ *ycf*1 in *Alnus cremastogyne*, 3 in *Ostryopsis davidiana* (MF375337), and 29 in *Alnus alnobetula* (MF136498), *Ostryopsis intermedia* (MG386376), and *Ostryopsis nobilis* (MG386378). The gene *ycf*1 spanned the IRa/SSC border in all plastomes, with the length of the *ycf*1 gene locating in the IRa region varying from 1,158 bp to 2,731 bp. The IRa/LSC boundary of 30 plastomes lay between the *rpl*2 and *trn*H genes, while that of *Corylus avellana* (MN082371) was uniquely situated between *trn*H and *rps*19 genes.

### Phylogenetic analyses

**Fig. 3:**
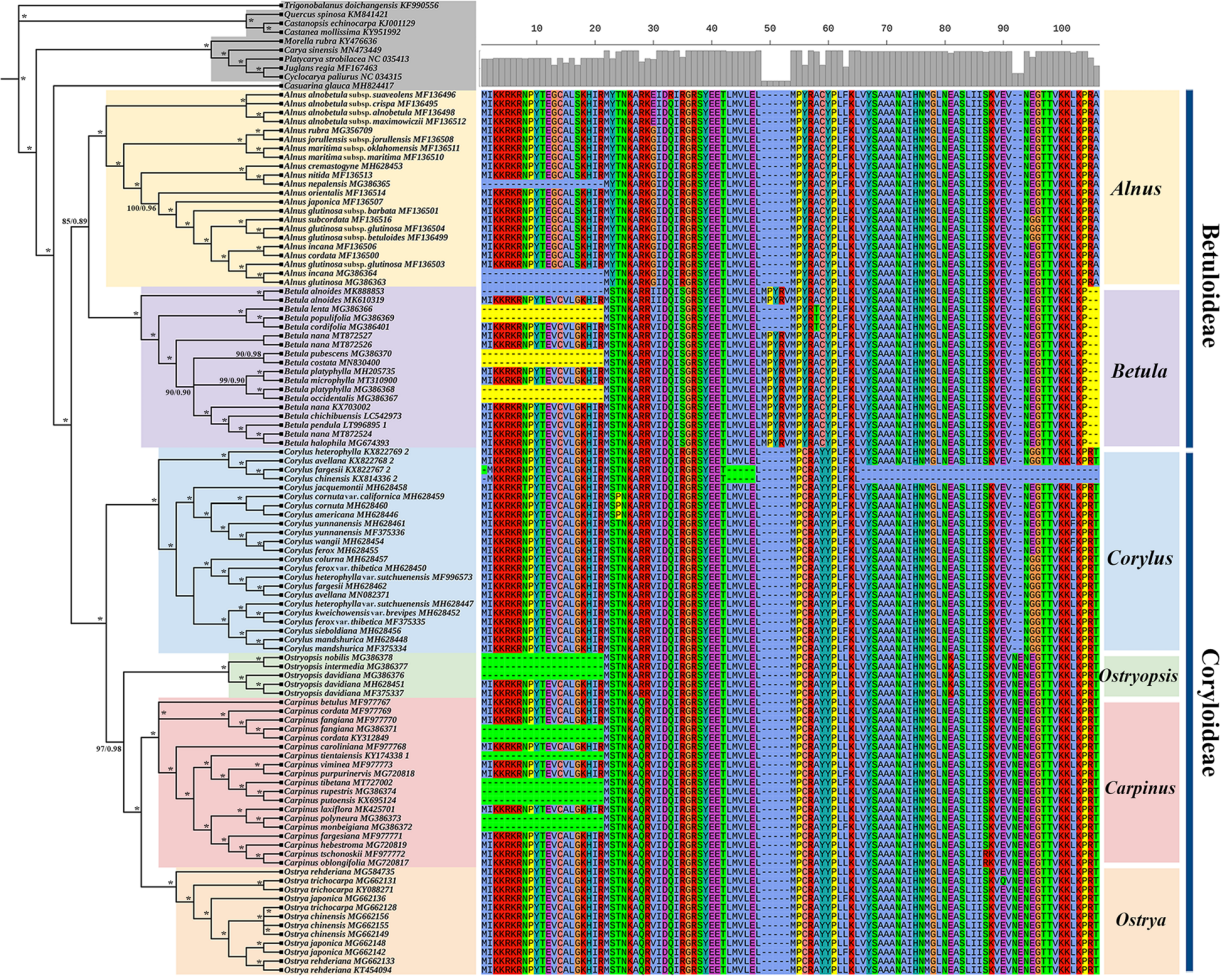
Phylogeny of Betulaceae based on CDS data and amino acid alignment of *rpl*22 gene. **A** Phylogenetic inference inferred by Maximum Likelihood (ML) and Bayesian inference (BI) analyses. BS and PP values are presented on the branches. Asterisks represent 100/1.0 support values. Major genera of Betulaceae are indicated by different colors. **B** Among these coding hotspots with nucleotide length greater than 200 bp, *rpl*22 showed the highest variation rate whose amino acid alignment is displayed on the right

Four datasets, i.e., protein-coding regions (CDS), non-coding regions (CNS), whole plastomes (WP), and divergence hotspots (DH), were subjected to phylogenetic analyses to test whether different data characters were responsible for any changes observed in support and resolution of the integrated phylogenies. Data characteristics and best-fit models for Maximum likelihood (ML) and Bayesian inference (BI) analyses were presented in Table S4. ML and BI analyses of each dataset generated almost congruent topologies with generally high bootstrap support (BS) and posterior probability (PP) (Figs. 3A and S2, S3, S4). In the CDS phylogeny, monophyly of each genus was highly supported, of which *Alnus* and *Betula* were resolved as sister groups (BS/PP = 85/0.89) and formed the subfamily Betuloideae. *Corylus*, *Ostryopsis*, *Carpinus*, and *Ostrya* were included in the subfamily Coryloideae, with *Corylus* located at the stem of the phylogeny to form the tribe Coryleae (BS/PP = 100/1) and the other three genera formed the tribe Carpineae (BS/PP = 97/0.98). Within Carpineae, *Ostryopsis* was placed at the basal position and constituted sister group to *Carpinus*-*Ostrya*, while the latter two showed a well-supported sister relationship (BS/PP = 100/1) (Fig. 3A). The phylogenies of the CNS and WP datasets displayed highly identical topologies that supported the monophyly of four genera (*Alnus*, *Betula*, *Corylus*, and *Ostryopsis*) and the paraphyly between *Carpinus* and *Ostrya*. *Ostrya trichocarpa* was found to locate at the stem of *Carpinus*, whereas *Carpinus hebestroma*, *C. oblongifolia*, *C. purpurinervis*, and *C. cordata* were situated in the basal of *Ostrya* (Figs. S2 and S3). The DH phylogeny showed similar intergeneric relationships to those of CNS and WP datasets with the exception that *Ostrya trichocarpa* was located at the basal of the above four *Carpinus* species (Fig. S4)*.*

### Divergence time estimation

**Fig. 4:**
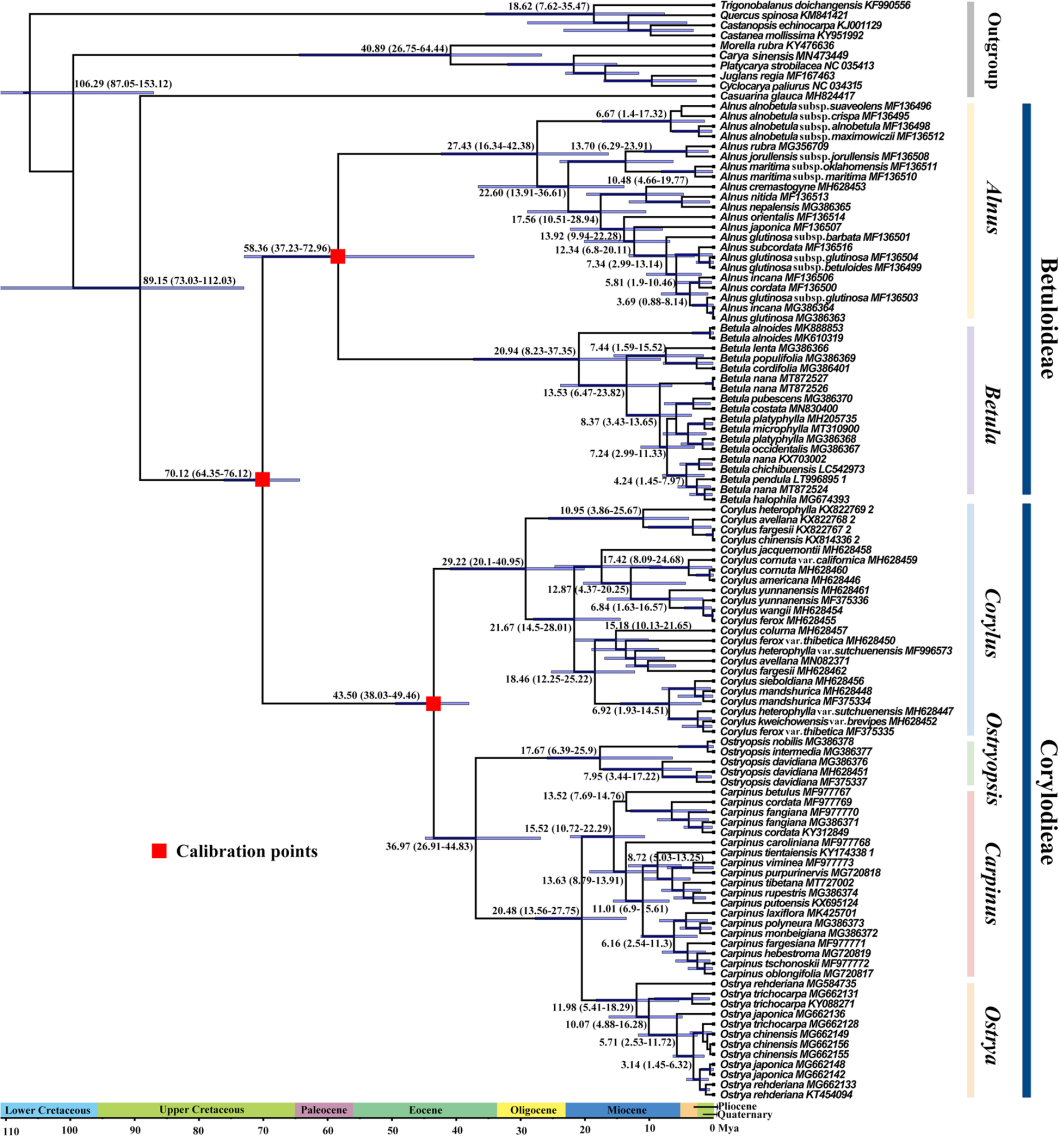
BEAST chronogram of divergence times for Betulaceae based on CDS data. Fossil calibrations are indicated by red boxes. Numbers above the tree branches represent median divergent ages and 95% HPD intervals. The blue bars represent the 95% highest posterior density of node ages

Molecular dating analysis based on CDS phylogeny indicated that the stem group of Betulaceae occurred during the upper Cretaceous (~ 89.15 Ma, 95% HPD = 73.03–112.03 Ma) (Table 2; Fig. 4). The crown age of Betulaceae and the split of Betuloideae and Coryloideae dated back to the Cretaceous-Paleogene boundary (~ 70.12 Ma, 95% HPD = 64.35–76.12 Ma). Within Betuloideae, *Alnus* and *Betula* diverged from each other shortly after the formation of the subfamily, approximately at 58.36 Ma (95% HPD = 37.23–72.96 Ma) during the Selandian age of the Paleocene. Within Coryloideae, *Corylus* and *Ostryopsis* successively diverged from the ancestral group in the middle (~ 43.50 Ma) and late Eocene (~ 36.97 Ma), respectively, while the sister genera *Carpinus* and *Ostrya* diverged from each other in the early Miocene (~ 20.48 Ma, 95% HPD = 13.56–27.75 Ma). The internal divergence within each genus occurred from the early Oligocene (~ 29.22 Ma) to the middle Miocene (~ 11.98 Ma), with *Corylus* diversifying the earliest and *Ostrya* the latest.

**Table 2:** Estimated divergence times for main clades within Betulaceae

Clades	Stem age (Ma)	95% HPD	Crown age (Ma)	95% HPD
Betulaceae	89.15	112.03–73.03	70.12	76.12–64.35
Subfam. Betuloideae	70.12	76.12–64.35	58.36	72.96–37.23
*Alnus*	58.36	72.96–37.23	27.43	42.38–16.34
*Betula*	58.36	72.96–37.23	20.94	37.35–8.23
Subfam. Coryloideae	70.12	76.12–64.35	43.50	49.46–38.03
*Corylus*	43.50	49.46–38.03	29.22	40.95–20.10
*Ostryopsis*	36.97	44.83–26.91	17.67	25.90–6.39
*Carpinus*	20.48	27.75–13.56	15.52	22.29–10.72
*Ostrya*	20.48	27.75–13.56	11.98	18.29–5.41

### Ancestral area reconstruction

**Fig. 5:**
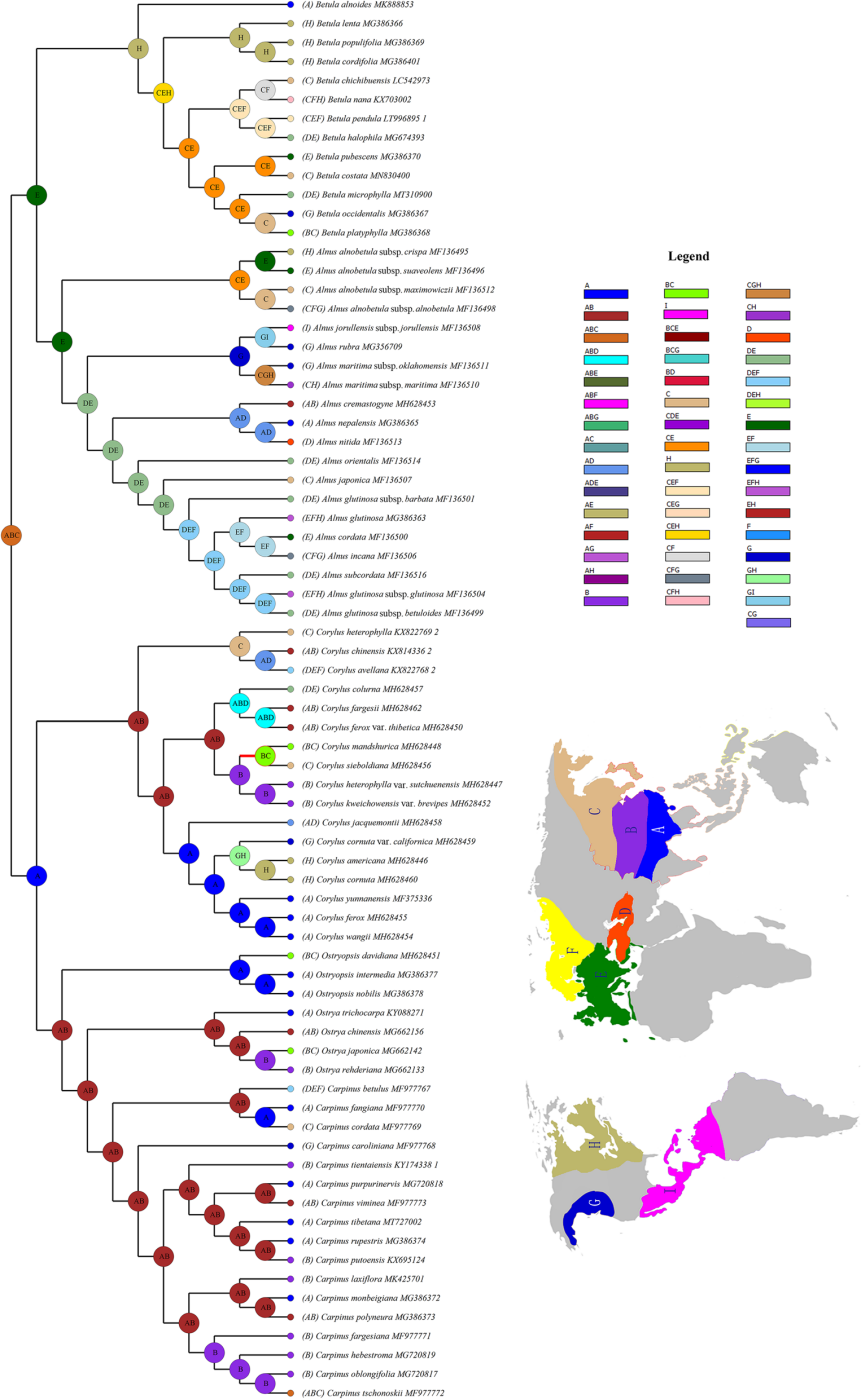
Ancestral area reconstruction based on the likelihood implementation of Bayesian inference for discrete Areas (BAYAREALIKE). Current distributions are indicated before the species names. The inserted map shows the contemporary distribution of Betulaceae species, covering nine major floristic divisions (**A-I**). Numbers and colors in the legend refer to extant and possible ancestral areas, and combinations of these

The likelihood implementation of Bayesian inference for discrete areas (BAYAREALIKE) reconstructed East Asia (ABC) as the ancestral area for the most recent common ancestor (MRCA) of Betulaceae (Fig. 5), although the exact subareas were not specified. In situ diversification of Betulaceae ancestors led to the formation of Coryloideae crown groups in southwestern East Asia (A), while westward dispersal resulted in the occurrence of Betuloideae crown group in southern Europe and the Mediterranean coast (E). Within Coryloideae, long-distance dispersals from A to E and North America (G, H), and subsequent vicariance events contributed to the intercontinental disjunction at the genus level. However, this pattern was not evident in *Carpinus* and *Ostrya* due to the limited sampling of non-Asian representatives. Within Betuloideae, E and eastern North America (H) were revealed as ancestral areas for *Alnus* and *Betula*, respectively. Likewise, long-distance dispersals from original centers to other parts were also observed, e.g., E to Central Asia (D) and then to A and central and eastern China (B); H to northern Europe (F) and then to E and D; as well as mutual exchanges between E and H. Overall, the connection/fracture of three important paths, i.e., North Atlantic Land Bridge, Beringian Land Bridge, and Mediterranean-eastern Himalayas/western China corridor, have played important roles in the intercontinental disjunction of Betulaceae.

### Morphological characters evolution

**Fig. 6:**
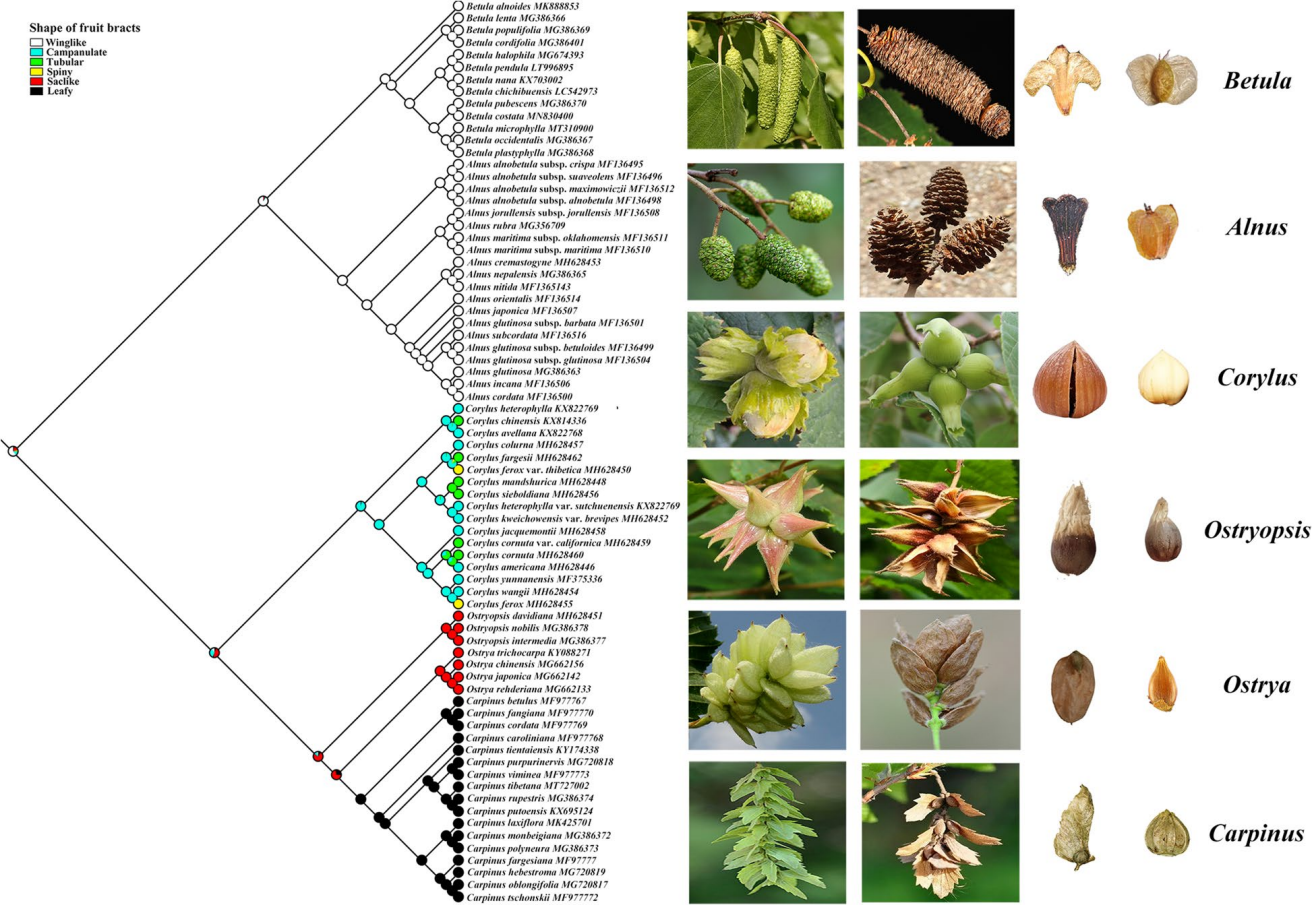
Ancestral state reconstruction of shape of fruit bracts. The images on the right show the typical characteristics of infructescence, fruits, and bracts for each genus, respectively

Ancestral states for 14 morphological characters are summarized in Fig. 6 and Figs. S5, S6, S7, including five flower characters (1–5), three anatomical characters (6–8), three leaf characters (9–11), and three fruit characters (12–14). For flower characters, it is unambiguously that the ancestor of Betulaceae had bisexual inflorescence, raceme infructescence, staminate perianth or pistilloide in male floret present, separated thecae and partly divided filaments. The aggregated infructescence seems to have evolved from raceme infructescence and then reversed in *Corylus* and *Ostryopsis* (Fig. S5), while the other characters have altered their states in parallel in different lineages. For anatomical characters, their ancestral states (e.g., scalariform vessel perforation, present tracheids, and absent tyloses) have evolved independently in different genera (Fig. S6). Regarding leaf characters, the presence of stomatal apparatus and embedded glands on leaves, and compound teeth were inferred as ancestral states, with embedded glands absent in subfamily Coryloideae and then inverted in *Ostryopsis* (Fig. S7). As for fruit characters, the ancestral states of winged diaspore and epigeal seed germination retained in all the extant genera except *Corylus*, whereas the shape of fruit bracts have evolved from winglike into multiple forms, especially in *Corylus* (Fig. 6).

## Discussion

### Plastome structural evolution of Betulaceae

Previous researches have suggested that the plastome size of angiosperms ranges from 107 kb in Pinaceae to 218 kb in Geraniaceae, and the size of the IR region is 20–30 kb [27, 28]. Our results showed that the plastomes of Betulaceae were located at the larger end of the angiosperm organelle genome, with Betuloideae having relatively larger genome (*Alnus*: ~ 160,690 bp; *Betula*: ~ 160,529 bp). By contrast, Coryloideae especially tribe Carpineae evolved relatively smaller genome (*Ostryopsis*: ~ 159,630 bp; *Carpinus*: ~ 159,338 bp; *Ostrya*: ~ 159,230 bp), whereas the transitional *Corylus* owned medium plastome size (~ 160,094 bp). Contraction and expansion of IR regions are very common in the process of evolution, and has been proved to be an important source of plastome size variation [20, 22, 29]. In our research, however, insignificant length variation for IR was detected among Betulaceae plastomes, with species of *Corylus* and *Carpinus* expanded slightly (Fig. 2; Table S1). This is normal because the high conservation of the IR region is also crucial for the stability of plastome structure [30]. Correspondingly, the length variation of LSC or SSC regions could have contributed to the differences in genome size. For example, *Alnus* and *Betula* had larger LSC and SSC regions than other genera, and although *Ostrya* possessed the largest SSC region in the Coryloideae, its LSC region was the smallest (Table 1, Table S1). Comparable results were also discovered in other taxa, such as Apiales [20], *Eucalyptus* [31], *Ampelopsis* [32], and those early diverging eudicots [33]. Gene loss occurs frequently in plastomes, for instance, the genes *rpl*22, *rps*16, *rpl*23, *acc*D, *ycf*1, and *inf*A were utterly or partially lost in the plastomes of legumes [34], and *acc*D, *ycf*1 were entirely missed in Poaceae [35]. In Betulaceae, the gene content varied slightly among species (121–134), with major differences lying in the numbers of tRNA (24–32) and protein-coding genes (78–80) (Tables 1, S1 and S2). Particularly, we discovered that two genes (*inf*A and *ycf*15) were lost in most of the Betulaceae plastomes (Table S2). Despite the changes in gene content, Betulaceae plastomes were highly conserved in terms of genome structure with only trivial IR expansion detected (Figs. 2 and S1). In addition, synteny analysis demonstrated that IR regions were more conservative than two SC regions, which is accordance with the conclusion that the accumulation of point mutations in the IR region is slower than the SC regions [36].

Plastome comparative genomics has been confirmed to facilitate the development of divergence hotspots which can be used for species delimitation and phylogenetic research of different levels [37, 38]. Relevant researches have shown that some coding genes of plastomes were efficient in resolving complex phylogenetic relationships of special plant taxa, for instance, *ndh*K, *psa*I and *rpl*22 in *Allium* [17], *psa*I, *pet*B, and *rps*16 in *Notopterygium* [39]. Furthermore, more studies revealed that non-coding regions were more variable than coding regions, and had higher resolution in species identification of related groups, e.g., *trn*T*-trn*L, *pet*D*-rpo*A and *ycf*4*-cem*A displayed apparent divergence in Veroniceae species [40], while *rpl*32*-trn*L, *rpo*B*-trn*C, *psa*C*-ndh*E, and *clp*P*-psb*B were highly variable in *Phalaenopsis* species [41]. Two widely used plastome markers, *rbc*L and *mat*K have been revealed to have limited resolution in previous phylogenetic studies of Betulaceae [2, 15, 42], conforming to their low nucleotide variation as shown in our research (*P*_*rbc*L_ = 0.0088, *P*_*mat*K_ = 0.0092) (Table S3). In the present study, both nucleotide diversity and mVISTA analysis revealed that the variation level of non-coding regions was significantly higher than coding regions (Figs. 1 and S1), which is consistent with previous results of most angiosperms [17, 37]. Correspondingly, we identified five non-coding hotspots (*P*_i_ > 0.035) that have not been reported in previous studies, i.e., *trn*T_GGT-*psb*D, *trn*E_TTC-*trn*T_GGT, *ndh*C-*trn*V_UAC, *trn*H-GTG_*psb*A, and *ycf*4_*cem*A; simultaneously, five coding genes, i.e., *psa*I, *ycf*1, *rpl*22, *psa*J, and *cem*A, exhibited higher diversity (*P*_i_ > 0.02) than other genes (Fig. 1; Table S3). Ten divergence hotspots can serve as candidate DNA barcodes for inferring phylogenetic relationships and intergeneric divergence of Betulaceae.

### Phylogenetic implications of plastome-scale dataset

Previous studies based on molecular and morphological data have contributed to an improved grasp on the taxonomy and intergeneric relationships of Betulaceae. Nevertheless, wide controversies still existed in terms of genera delimitation and their evolutionary relationships. Within Betuloideae, *Betula* and *Alnus* were either treated as sister monophyly [2, 4, 12] or assigned as paraphyly in which *Alnus* lay in the basal position [14, 43]. The situation was more complicated within Coryloideae, especially the phylogenetic placement of *Ostryopsis* as well as the generic relationships between *Carpinus* and *Ostrya*. *Ostryopsis* was located at the basal of the *Carpinus*-*Ostrya* clade based on nuclear ITS [2, 12, 16], chloroplast *rbc*L [2] and morphology [2, 8] data, while the genus formed sister clade with *Corylus* in the multi-gene (ITS, *mat*R, *rbc*L, *trn*L) partitioned phylogeny [44] and the *mat*K tree [15]. Inferred from ITS phylogeny, *Carpinus* and *Ostrya* were either mutual monophyly [2], or *Carpinus* was treated as paraphyly with *Ostrya* inserted within it [16]. Besides, the combined chloroplast fragments (*psb*A-*trn*H, *trn*L-*trn*F, and *mat*K) inferred *Ostrya* as paraphyly [42]. Such kind of phylogenetic controversies can result from a variety of factors, including incomplete taxa sampling, limited sequence variation, and heterogeneous evolutionary rate among genes [45, 46]. In this research, we obtained the most extensively sampled and well-resolved phylogeny for Betulaceae based on pan-plastome data. Our trees presented an essential improvement in internode resolution compared with previous phylogenetic inferences. With the results, some long-standing controversies have been clarified. The phylogenomic backbone inferred from all datasets (CDS, CNS, WP, and DH) highly supported the division of Betulaceae into Betuloideae and Coryloideae, and reciprocal monophyly between *Alnus* and *Betula* (Figs. 3A and S2, S3, S4). Particularly, the phylogenetic position of *Ostryopsis* was well established, with *Corylus*, *Ostryopsis*, *Carpinus*-*Ostrya* forming successive sister lineages in Coryloideae. The evolutionary relationships between *Carpinus* and *Ostrya* were also illuminated. The two genera formed mutual paraphyly in three phylogenies (CNS, WP, and DH), but were well supported as reciprocal monophyly in the CDS tree. This phylogenetic conflict actually reflects the heterogeneity of evolutionary rate in different genes and structural regions. Non-coding regions, especially intergenic spacers, have higher evolutionary rate than protein-coding regions, while divergence hotspots represent the most variable level in the whole plastome. Hence, despite high-resolution phylogenies inferred from these concatenated super matrices, various evolutionary rates in different regions could inevitably lead to rampant phylogenetic discordance at all levels of angiosperm phylogeny [46].

Just as the paraphyletic relationships between *Carpinus* and *Ostrya*, non-monophyly is relatively common when multiple accessions of each taxon are applied in phylogenetic studies [47]. Such non-monophyly usually reflects two genealogical processes, i.e., incomplete lineage sorting and introgressive hybridization, which are hard to distinguish due to their similar phylogenetic signature [48]. The relatively late differentiation implies that incomplete sorting of ancestral polymorphisms may play an important role in maintaining the paraphyletic status of the two genera. In turn, incomplete lineage sorting could result from rapid radiation during early diversification. Accordingly, it is not surprising that previous single-gene studies or even our phylogenomic research have placed *Carpinus* and *Ostrya* into different phylogenetic arrangements. Hybridization/introgression can result in horizontal transfer of maternally inherited plastomes into introgressive species when closely related species are sympatric distribution and reproductive compatibility [49]. Introgression-induced chloroplast capture has been proved as a particular mechanism to generate distorted phylogenetic relationships, in which introgressive taxa always present typical geographic clustering [50]. In Betulaceae, natural hybridization is frequently found to occur within a genus [50–52], but few has been reported between two different genera. Recently, homploid hybrid speciation between ancestors of *Carpinus* and *Ostrya* was revealed by genetic evidence [53], indicating that intergeneric hybridization could be achieved during the initial stages of differentiation. Similar cases have been reported in other plant taxa, e.g., a common ornamental plant known as “ × *Heucherella*” in the nursery trade stems from the hybridization between *Heuchera* and *Tiarella* [54]. In our plastome phylogenetics, the nonrandom phylogenetic clustering (reciprocal basal lineages of each other) among *Carpinus* and *Ostrya* species (Figs. S2 and S3) may further corroborate the hypothesis of chloroplast capture caused by intergeneric introgressive hybridization. However, the geographic clustering of intergeneric species is not obvious probably because ancient chloroplast capture between genera may be blurred by frequent plastome transfer among intrageneric species. In total, our phylogenies are relatively robust and can well reflect the actual evolutionary relationships of the family.

### Diversification and biogeographic history

A well-resolved phylogeny, extensive taxon sampling, and reliable fossil calibrations have been shown to be crucial for estimating divergence times and biogeographic history [55–57]. Although relatively rich fossils for Betulaceae have encouraged molecular dating studies, no agreement has been reached, mainly due to different sampling scales, molecular markers, and fossil calibrations [4, 12, 44, 58]. In this research, utilizing an extensively sampled, well-resolved phylogeny, and reliable fossil calibrations, we re-evaluated the divergence history of major lineages within Betulaceae (Fig. 4; Table 2). Our estimated ages were much younger than those of Forest et al. in many clades, especially the crown group of Betulaceae (~ 119.3 Ma), Betuloideae (~ 109.0 Ma), and Coryloideae (~ 70.2 Ma) [12]. By contrast, estimates for most basal nodes were nearly ten million years older than those of Grimm and Renner when they calibrated the stem lineage of Betulaceae with a 71 Ma-old flower fossil [4]. Comparably, our results were compatible with a recent study which also applied whole plastomes to infer the divergence time within Betulaceae [58]. However, their sampling only involved a few species in each genus and their basal lineages were largely missed, which may have led to the under-estimation of the crown ages of extant genera. Overall, our estimates are relatively robust and reliable. On one hand, the phylogenetic frame was constructed based on pan-plastome data that was more informative than single or several fragments so that less bias was introduced. On the other hand, the estimated ages for major nodes were congruent with reliable fossil evidences. For instance, the earliest pollen fossils belonging to the complex groups of *Betula* and *Alnus* were confirmed to have occurred in the Santonian/Campanian of Japan and North America [59], which were coetaneous with the 71 Ma-old flower fossils used in our calibration. They jointly indicated that the crown of Betulaceae occurred during the upper Cretaceous, as inferred by Xiang et al. (~ 74.99 Ma) and the present study (~ 70.12 Ma) [60]. Likewise, fruit fossils from the Republic flora of northeastern Washington were the oldest known occurrences of two extant genera (*Carpinus* and *Corylus*) [10], indicating a middle-Eocene origin of Coryloideae. Coincidentally, our estimates revealed an almost identical age for the crown group of Coryloideae (43.50 Ma). Therefore, our results present a more realistic scenario for the divergence history of Betulaceae.

Betulaceae is believed to have originated in central/western China in the late Cretaceous (∼70 Ma) [61]. The assumption is supported by extensive fossil records and the fact that all six extant genera and almost one-third of the species of Betulaceae are native to this region. This biogeographic origin is further evidenced by ancestral area reconstructions and divergence age estimates conducted in the present study, that is, the MRCA of Betulaceae occurred in East Asia at the Cretaceous-Paleocene boundary (~ 70.12 Ma) (Figs. 4 and 5). During this period, a multitude of species have experienced rapid diversification on account of global cooling and the emergence of new habitats [62, 63]. Hence, it is very likely that adaptive radiation triggered by environmental changes has contributed to the initial differentiation of Betulaceae. Despite the East Asian origin of Betulaceae, both in situ and allopatric diversification were detected, with A and E inferred as the original center for the crown groups of Coryloideae and Betuloideae, respectively. Apparently, the preexisting corridor between eastern Himalayas/western China and European-Mediterranean region has facilitated ancient intercontinental dispersal, thus generating the European ancestors of Betuloideae. The strong floristic connection between East Asia and Europe has also been demonstrated in previous biogeographic studies [50, 64]. The intergeneric divergence within Betuloideae (~ 58.36 Ma) was relatively earlier than that of Coryloideae, which was probably caused by contrasting intercontinental habitat differences between *Alnus* and *Betula*, i.e., E and H. The favorable environments (warm and humid climate) during the middle Paleocene may have further contributed to their distribution around the northern hemisphere [65]. Particularly, rich macrofossil records of *Alnus-*like species indicated that the ancestors of this genus have dominated the flora of North America since the Paleocene [66]. By comparison, the ancestors of the four genera (*Corylus*, *Ostryopsis*, *Carpinus*, and *Ostrya*) within Coryloideae were sympatric distribution in East Asia, and the relatively homogeneous habitats could have delayed their intergeneric divergence, roughly in the middle Eocene (~ 43.50 Ma). With the global cooling at that time, adaptive radiation should have played an essential role in promoting this divergence processes. From the late Eocene to the Pliocene, a series of geoclimatic events, such as the uplift of the Qinghai-Tibet Plateau, the formation of the Asia monsoon, as well as the Quaternary glaciation cycles [67–69], may have driven the lineage diversification and radiative speciation within each genus. Notably, the connection/fracture of three important paths, i.e., North Atlantic Land Bridge, Beringian Land Bridge, and Mediterranean-eastern Himalayas/western China corridor, have played important roles in the intercontinental disjunction of Betulaceae.

### Evolution of key characters of Betulaceae

The acquisition of new morphological traits has influenced the diversification of various plant groups [70, 71]. The six genera within Betulaceae are typical representatives to increase the number of such cases. State reconstructions revealed that bisexual and raceme infructescence with staminate perianth and pistilloide in male floret were the most likely ancestral traits for Betulaceae (Fig. S5). Most of these flower characters have changed their states only once during evolution in different genera except that raceme infructescence evolved into aggregated infructescence and then reversed in *Corylus* and *Ostryopsis*. Generally, flower characters for the six genera have a tendency towards simplification. In recent studies, the hypothesis that the evolution of floral variation is driven by pollinator transfer has been confirmed by phylogenetic evidences [22, 72, 73]. In Betulaceae, two types of pollinators, namely, anemophilous and entomophilous pollination, were recognized. Although both ways were equally important for pollination of Betulaceae in the early Tertiary, enhancement of fecundity and pollination efficiency gradually promoted insect-pollination as the dominant mode [2]. Accordingly, each genus has evolved corresponding floral features independently. Currently, few studies have been carried out on the interaction between Betulaceae plants and pollinators. In addition to overall survey on plant-pollinator interaction, relevant ecological factors should also be considered to better clarify the evolution of flower traits of Betulaceae plants.

Anatomical and leaf characters were important traits for taxonomic and systematic consideration within Betulaceae. And, it becomes clear that some key characters well reflect the evolutionary order among the six genera (Figs. S6 and S7). For example, *Carpinus* and *Ostrya*, the two youngest sister genera in the family, coevolved simple vessel perforations and tyloses in anatomical structure and degraded glands on leaves, of which *Carpinus* species further obtained typical stomatal apparatus in their leaves. By comparison, two primitive genera *Alnus* and *Betula* retained most of the ancestral states of Betulaceae, including scalariform perforations, distinct tracheid, absent tyloses and stomatal apparatus, and embedded glands on leaves. *Corylus* and *Ostryopsis* seem in some aspects to be transitional between Coryloideae and Betuloideae. On one hand, the two share some characters with *Alnus* or *Betula*, such as scalariform perforations, absent tyloses and stomatal apparatus. On the other hand, they display characters in common with *Ostrya* or *Carpinus*, including degraded tracheid, and present pistilloide in male floret. Notably, we reveal that some characters are parallelly evolved even between sister genera, including the cases of pistilloide in male floret, thecae and filaments, stomatal apparatus, and leaf teeth between *Carpinus* and *Ostrya*, *Alnus* and *Betula*. This indicates that divergent evolution of multiple characters has involved in the divergence among sister lineages.

Evolution of fruit types and their dispersal modes is recognized as important drivers of angiosperms diversification [74, 75]. Despite diverse fruit and diaspore types in the plant kingdom we observe today, it has taken a long time to finish this evolutionary process. In the Cretaceous, angiosperms were still dominated by small fruits, and abiotic dispersal was the mainstream. During the Eocene, average fruit size increased sharply, and biotic dispersal by vertebrates has become much more prevalent. In the late Tertiary, average fruit size tended to decrease as the climate cooled and vegetation opened [76]. In the present study, divergence time estimation and ancestral character reconstruction reveal consistent trends between fruit character evolution and historical geo-climatic changes (Figs. 4 and 5). First, Betulaceae ancestors were inferred to have small winglike fruits and winged diaspores, corresponding to the ancestral states of most angiosperms in the Cretaceous. Thereafter, the two primitive genera *Alnus* and *Betula* retained these ancestral states in subsequent evolution. Second, *Corylus* exclusively evolved prominent large nuts in the Eocene, conforming with the conspicuous increase in seed and fruit sizes in the early Tertiary. Especially, a variety of bract shapes have occurred in *Corylus*, with most species owning campanulate or tubular bracts and a few species having spiny bract. Finally, *Ostryopsis* and *Ostrya* parallelly retrieved small nuts enclosed by saclike bracts, while *Carpinus* further obtained small leafy nuts independently, coinciding with the reversal of fruit size in the late Tertiary. Hence, adaptive differences among genera at different historical stages may be the primary driving force for the evolution of fruit size and dispersal mode.

## Conclusion

Our research documented a comprehensive plastome feature for Betulaceae at species level, and represented the most robust phylogenetic inference to date. Comparative genomics showed that Betulaceae plastomes were highly conserved in genome size, gene order, and structure, although specific variations such as gene loss and IR boundary shifts were revealed. Six coding regions (*P*_i_ > 0.02) and five non-coding regions (*P*_i_ > 0.035) were identified as candidate DNA barcodes for phylogenetic analyses and taxonomic research. Furthermore, our phylogenomic studies clarified some unsolved phylogenetic issues, e.g., reciprocal monophyly between *Betula* and *Alnus*, successive sister relationships among *Corylus*, *Ostryopsis*, and *Carpinus*-*Ostrya*, and mutual paraphyly between *Ostrya* and *Carpinus*. Based on robust phylogenetic framework, we inferred that Betulaceae ancestors originated from East Asia during the upper Cretaceous; dispersals and subsequent vicariance accompanied by historical environment changes contributed to its diversification and intercontinental disjunction. Ancestral state reconstruction indicated the acquisition of many taxonomic characters was actually the results of parallel or reversal evolution. Overall, the results provide new insights into the plastome structural variation, phylogenetic relationships among major lineages, and simultaneously help to elucidate the diversification history and evolution of Betulaceae.

## Methods

### Taxon sampling and DNA isolation

A total of 109 plastomes representing Betulaceae and allied families were included in this study. Ninety-nine plastomes from the six genera of Betulaceae were selected, including *Alnus* (22, ca. 30 spp.), *Betula* (18, ca. 50 spp.), *Corylus* (23, ca. 20 spp.), *Ostryopsis* (5, 3 spp.), *Carpinus* (19, ca. 30 spp.), and *Ostrya* (12, ca. 8 spp.). Ten plastomes of allied families in Fagales were chosen as outgroup in the phylogenetic analysis. Among these 109 plastomes, sixteen were sequenced and assembled by our laboratory and the others were obtained from GenBank (Table S1). Leaves used in this study were either collected from natural populations in China or friendly provided as herbarium specimens by cooperative institutions. The formal identification of these plant materials was undertaken by Prof. Guixi Wang (Chinese Academy of Forestry). Voucher specimens were deposited in the non-wood forest laboratory of Research Institute of Forestry, Chinese Academy of Forestry, Beijing, China. Sampling information and herbarium vouchers were offered in Table 1. Genomic DNA of each sample was extracted from leaves (silica gel-dried or fresh materials) using DNeasy Plant Mini Kit (Qiagen, Beijing, China). The DNA quality and purity were evaluated using the Qubit Fluorometric Quantitation (Thermo Fisher, Scientific, USA) instrument and agarose gel electrophoresis.

### Plastome assembly and annotation

Library construction and paired-end sequencing were performed with Illumina HiSeq 2500-PE125 platform at Novogene (Beijing, China). The raw reads were checked using NGS QC Toolkit [77] with the following criteria: removing PCR duplicates and adapters; filtering reads with N over 10%; filtering reads with a mass value more than 40% and less than 10%. Based on these obtained high-quality reads, plastomes were assembled using MITObim v1.7 [78] with multiple Betulaceae plastomes as reference sequences. Small gaps existed in the assembled plastomes were filled and corrected by PCR-based sanger sequencing. PCR procedures and primers were provided in Table S5. These newly assembled plastomes were annotated employing both the program GeSeq [79] and DOGMA [80]. Draft annotations were further adjusted and verified through a BLAST alignment against the published Betulaceae plastomes. Besides, we renovated the annotations of previously published plastomes before using them in our study. All 16 newly obtained plastomes were submitted to GenBank with accession numbers provided in Table 1.

### Comparative genomics and structural analyses

To compare the structure variation and identify arrangement events across the family, comparative genomics of major lineages (31 representing plastomes) were performed under the Shuffle-LAGAN strategy in mVISTA [81] with the annotation of *Alnus alnobetula* as reference. The junction sites of four structural regions (IRA, LSC, SSC, IRB) and adjacent genes in these plastomes were ascertained using the online program IRSCOPE [82] to investigate the expansion or contraction of IRs. To explore variability among different regions (protein-coding regions and non-coding regions) for species identification and population genetics, nucleotide diversity (*P*_i_) of these regions was estimated with DnaSP 5.0 [83].

### Phylogenetic inference

Due to the heterogeneous evolutionary rate of different regions in plastome, we generated three datasets for phylogenetic analyses: protein-coding regions (CDS), non-coding regions (CNS), and whole plastomes (WP). The CDS and CNS sequences of all 109 plastomes were extracted with PhyloSuite v1.2.2 [84] and aligned separately using MAFFT v7.4 [85]. Then, these individual alignments were concatenated into supermatrices by PhyloSuite v1.2.2. We excluded ambiguously aligned sites from the three datasets, especially the WP dataset and the CNS dataset, using trimAl v1.2 with the automated1 model [86]. In addition, in order to evaluate the power of potential barcodes in phylogenetic inference, we also generated the fourth dataset (DH) by concatenating ten newly developed divergence hotspots obtained through comparative genomics analysis (Fig. 1; Table S3). Independent phylogenetic analyses were performed for each dataset (CDS, CNS, WP, and DH) using both Maximum likelihood (ML) and Bayesian inference (BI) strategies. The optimal nucleotide substitution models were calculated with the built-in ModelFinder program of PhyloSuite v1.2.2 under the Bayesian information criterion. The ML analysis was conducted by IQ-tree 1.63 [87] with support values evaluated by approximate likelihood-ratio test of 1,000 replicates and ultrafast bootstrapping of 5,000 replicates. The BI inference was implemented in MrBayes v3.26 [88]. Two independent chains (2 × 10^7^ generations for each chain) were conducted with starting from random trees. The sampling frequency was set as 1,000 generations and the first 25% of trees were discarded as burn-in. Stationarity was assumed when the average standard deviation of split frequencies < 0.01. The posterior probability (*PP*) values were estimated based on the remaining trees. FigTree v1.4.2 [89] was utilized to visualize the phylogeny.

### Divergence time estimation

Molecular dating analysis was performed with the Bayesian molecular-clock method in BEAST 1.84 [90] based on CDS data set. Three fossils were used to constrain the internal nodes: (1) According to the ancient flower fossils that represent an extinct lineage at the basal Betulaceae [91], 71.0 Ma was assigned as a mean age for the crown group of the family, and sigma of 3.0 was set for the normal prior distribution. (2) Based on the earliest pollen fossils for *Alnus* [92], we set a minimum age as 58.0 Ma to calibrate the split between *Betula* and *Alnus*. The prior was set as a log-normal distribution, with the offset 58.0, mean 1.0, and sigma 0.5. (3) From reports on the oldest fossils for *Palaeocarpinus*, *Carpinus*, and *Corylus* [10, 12], the most recent common ancestor of subfamily Coryloideae was constrained with an age between 37.0 Ma and 49.0 Ma. The prior was treated as normal distribution and sigma 3.0. BEAST was implemented using the uncorrelated log-normal relaxed clock and the GTR + G substitution model that was calculated by ModelFinder (Table S4). Yule process was selected as tree prior. Two independent MCMC simulations were conducted with each running 4.0 × 10^7^ generations and sampling every 1,000 generations. Convergence and stationarity of the results were checked by Tracer v1.7 [93]. Nodal heights and 95% highest posterior density intervals were summarized using TreeAnnotator v2.12 [94], with the first 25% trees treated as burn-in.

### Biogeographical inference

Ancestral area reconstruction was performed using the BioGeoBEARS plugin in RASP v4.3 [95]. A pruned time-calibrated tree including 74 taxa (one accession per species) inferred from BEAST analysis was used as the input tree. Due to lack of information on the ancestral distribution of outgroups, we also removed the outgroups from the tree in the biogeographic analysis. The likelihood implementation of Bayesian inference for discrete areas (BAYAREALIKE) was selected as the best biogeographical model by BioGeoBEARS according to the corrected Akaike information criterion (Table S6). All terminal taxa were assigned to nine geographic areas based on the distribution of species diversity and endemicity: (A) southwestern East Asia, (B) central and eastern China, (C) Northeast Asia, (D) Central Asia, (E) southern Europe and the Mediterranean coast, (F) northern Europe, (G) western North America, (H) eastern North America, (I) Central America. The maximum range size was set as three in that it is the maximum number of geographic areas where an extant genus occurs.

### Ancestral character state reconstruction

We explored character evolution for 14 morphological characters frequently used in the systematic and taxonomical studies of Betulaceae [2, 16, 44, 96], including characters of inflorescence, flowers, wood anatomy, leaves, and fruits. Information of these characters and their state scores for each species are provided in Table S7. Ancestral states were reconstructed using the one-parameter Markov k-state evolutionary model in Mesquite 3.51 [97]. For this analysis, a compiled tree (generated by the BEAST) containing 74 Betulaceae species was employed as the input tree. The corresponding characters were mapped onto the BI tree and the levels of homoplasy for each character were evaluated qualitatively.

## Supplementary Information

**Additional file 1: Figure S1.** Sequence identity plot of 31 Betulaceae plastomes using mVISTA. Gray arrows and thick black lines above the alignment indicate genes with their orientation and the position of the IRs, respectively. Different regions (LSC, IR, and SSC) are represented by different colors. **Figure S2.** Phylogeny of Betulaceae inferred from Maximum likelihood (ML) and Bayesian inference (BI) based on 109 whole plastomes (WP). BS and PP values are presented on the branches. Asterisks represent 100/1.0 support values. Major genera of Betulaceae are indicated by different colors. **Figure S3.** Phylogeny of Betulaceae inferred from Maximum likelihood (ML) and Bayesian inference (BI) based on non-coding sequences (CNS). BS and PP values are presented on the branches. Asterisks represent 100/1.0 support values. Major genera of Betulaceae are indicated by different colors. **Figure S4.** Phylogeny of Betulaceae inferred from Maximum likelihood (ML) and Bayesian inference (BI) based on divergence hotspots (DH). BS and PP values are presented on the branches. Asterisks represent 100/1.0 support values. Major genera of Betulaceae are indicated by different colors.

**Additional file 2: Table S1.** Accession numbers and structural features of all 93 plastomes obtained from GenBank. **Table S2.** Summary of protein-coding genes in all Betulaceae plastomes. **Table S3.** Statistics of nucleotide diversity for 78 coding genes and 68 non-coding regions. **Table S4.** Data characteristics and best-fit models for ML and BI phylogenetic analyses. **Table S5.** Primers used for gap closure in this study. **Table S6.** Results of model test used for biogeographic inference.

## Data Availability

All sequences described in this study are available in the GenBank repository under the accessions as summarized in Table 1 and Additional file 2: Table S1.
