# Supplementary material for: Plastome phylogenomics provide new perspective into the phylogeny and evolution of Betulaceae (Fagales)
Source: BMC Plant Biol. 2022 Dec 24;22:611. doi: 10.1186/s12870-022-03991-1 (PMC9789603; doi:10.1186/s12870-022-03991-1)
Supplement: Supplementary file 1 — Additional file 1: Figure S1. Sequence identity plot of 31 Betulaceae plastomes using mVISTA. Gray arrows and thick black lines above the alignment indicate genes with their orientation and the position of the IRs, respectively. Different regions (LSC, IR, and SSC) are represented by different colors. Figure S2. Phylogeny of Betulaceae inferred from Maximum likelihood (ML) and Bayesian inference (BI) based on 109 whole plastomes (WP). BS and PP values are presented on the branches. Asterisks represent 100/1.0 support values. Major genera of Betulaceae are indicated by different colors. Figure S3. Phylogeny of Betulaceae inferred from Maximum likelihood (ML) and Bayesian inference (BI) based on non-coding sequences (CNS). BS and PP values are presented on the branches. Asterisks represent 100/1.0 support values. Major genera of Betulaceae are indicated by different colors. Figure S4. Phylogeny of Betulaceae inferred from Maximum likelihood (ML) and Bayesian inference (BI) based on divergence hotspots (DH). BS and PP values are presented on the branches. Asterisks represent 100/1.0 support values. Major genera of Betulaceae are indicated by different colors. [file 12870_2022_3991_MOESM1_ESM.docx]

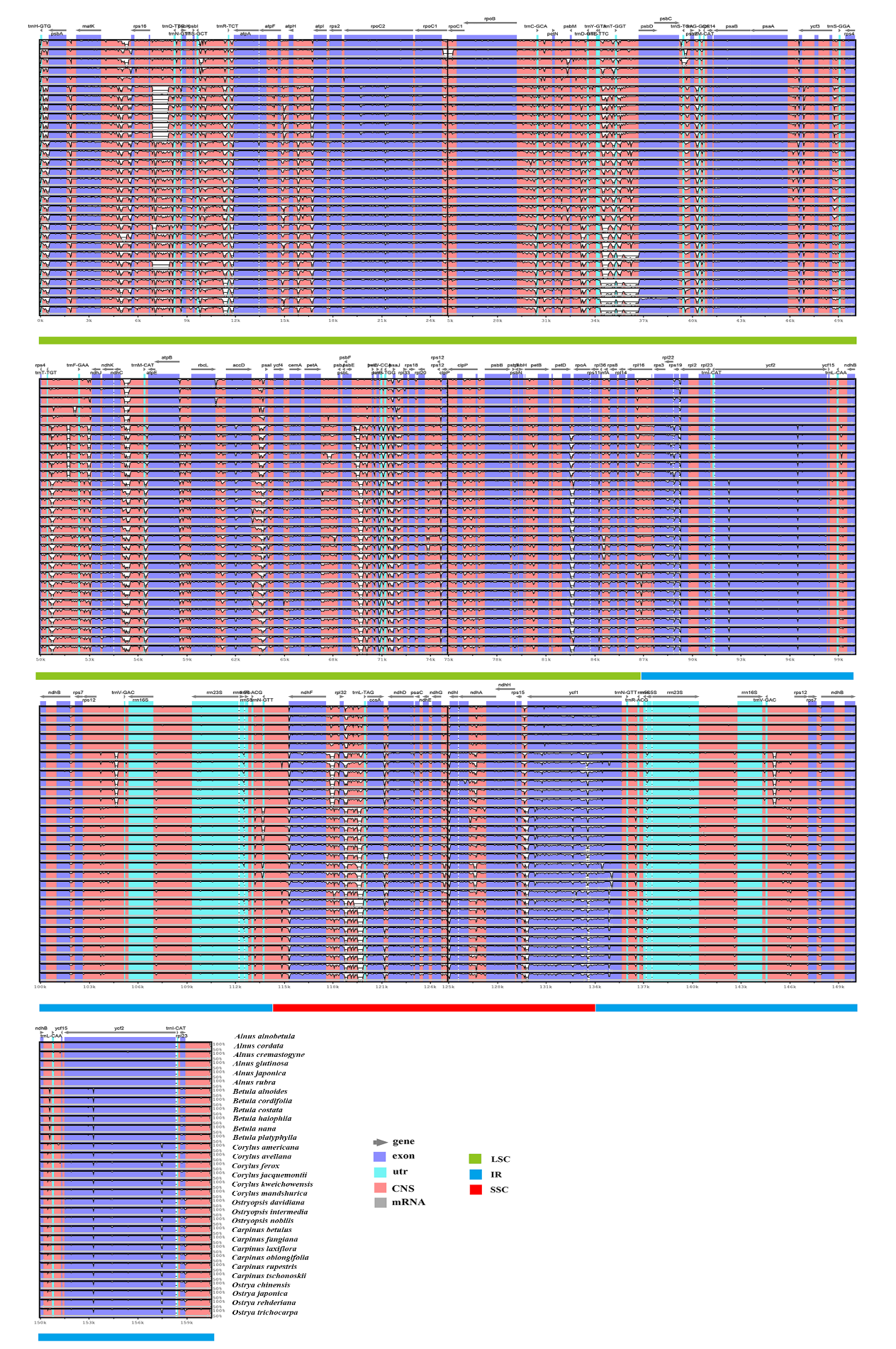


**Figure S1. Sequence identity plot of 31 Betulaceae plastomes using mVISTA.** Gray arrows and thick black lines above the alignment indicate genes with their orientation and the position of the IRs, respectively. Different regions (LSC, IR, and SSC) are represented by different colors.


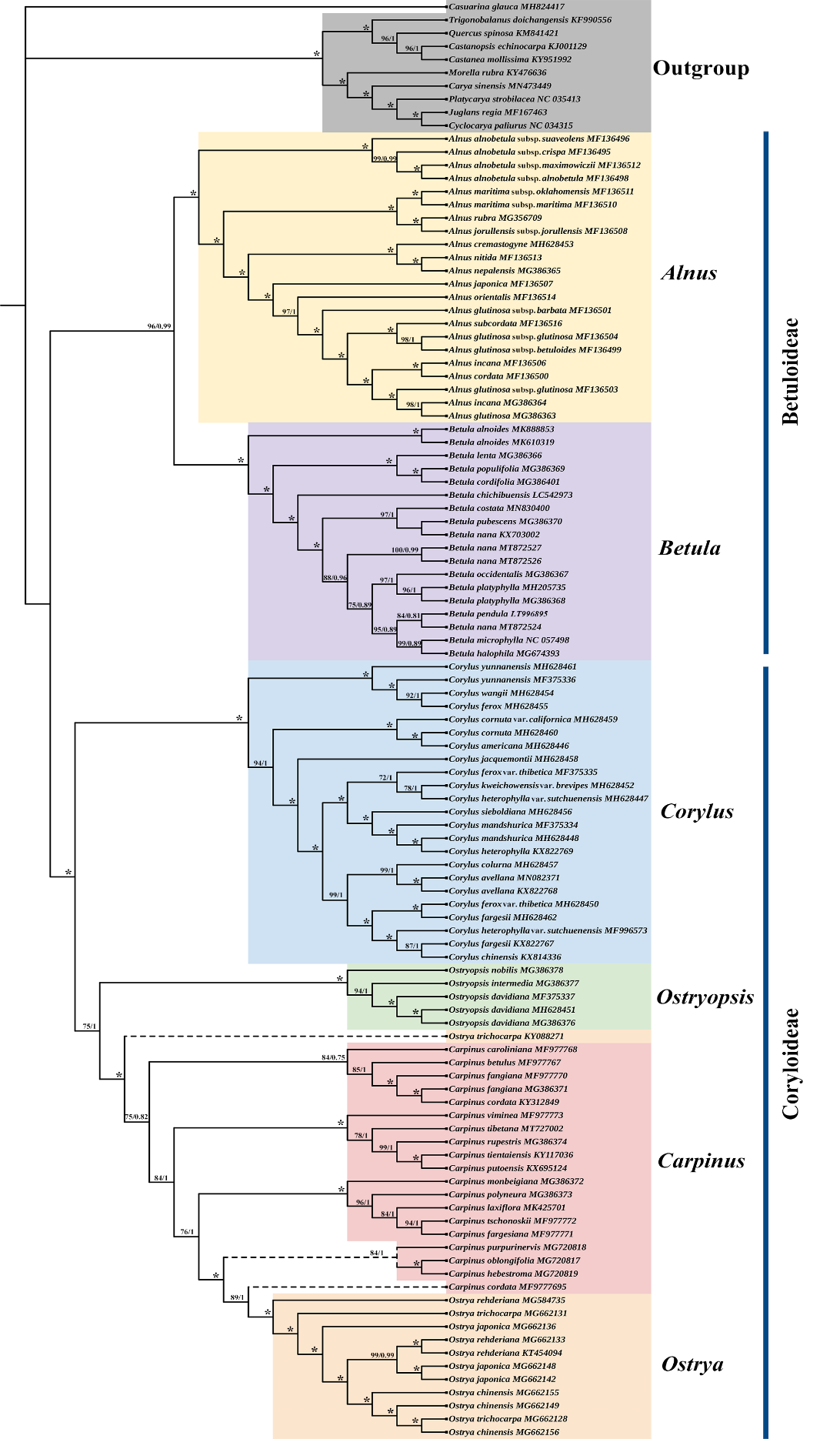


**Figure S2. Phylogeny of Betulaceae inferred from Maximum likelihood (ML) and Bayesian inference (BI) based on 109 whole plastomes (WP).** BS and PP values are presented on the branches. Asterisks represent 100/1.0 support values. Major genera of Betulaceae are indicated by different colors.


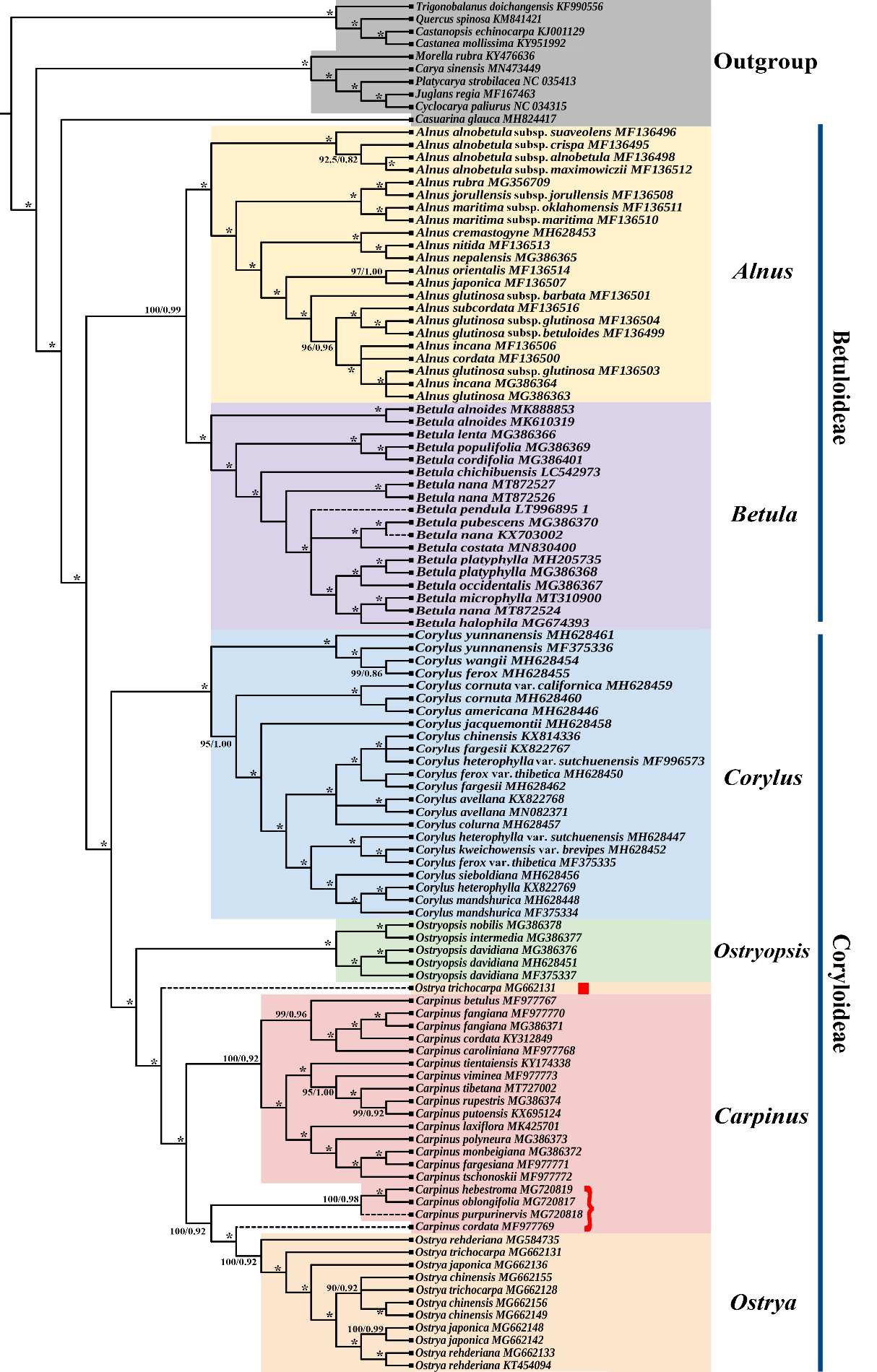


**Figure S3. Phylogeny of Betulaceae inferred from Maximum likelihood (ML) and Bayesian inference (BI) based on non-coding sequences (CNS).** BS and PP values are presented on the branches. Asterisks represent 100/1.0 support values. Major genera of Betulaceae are indicated by different colors.


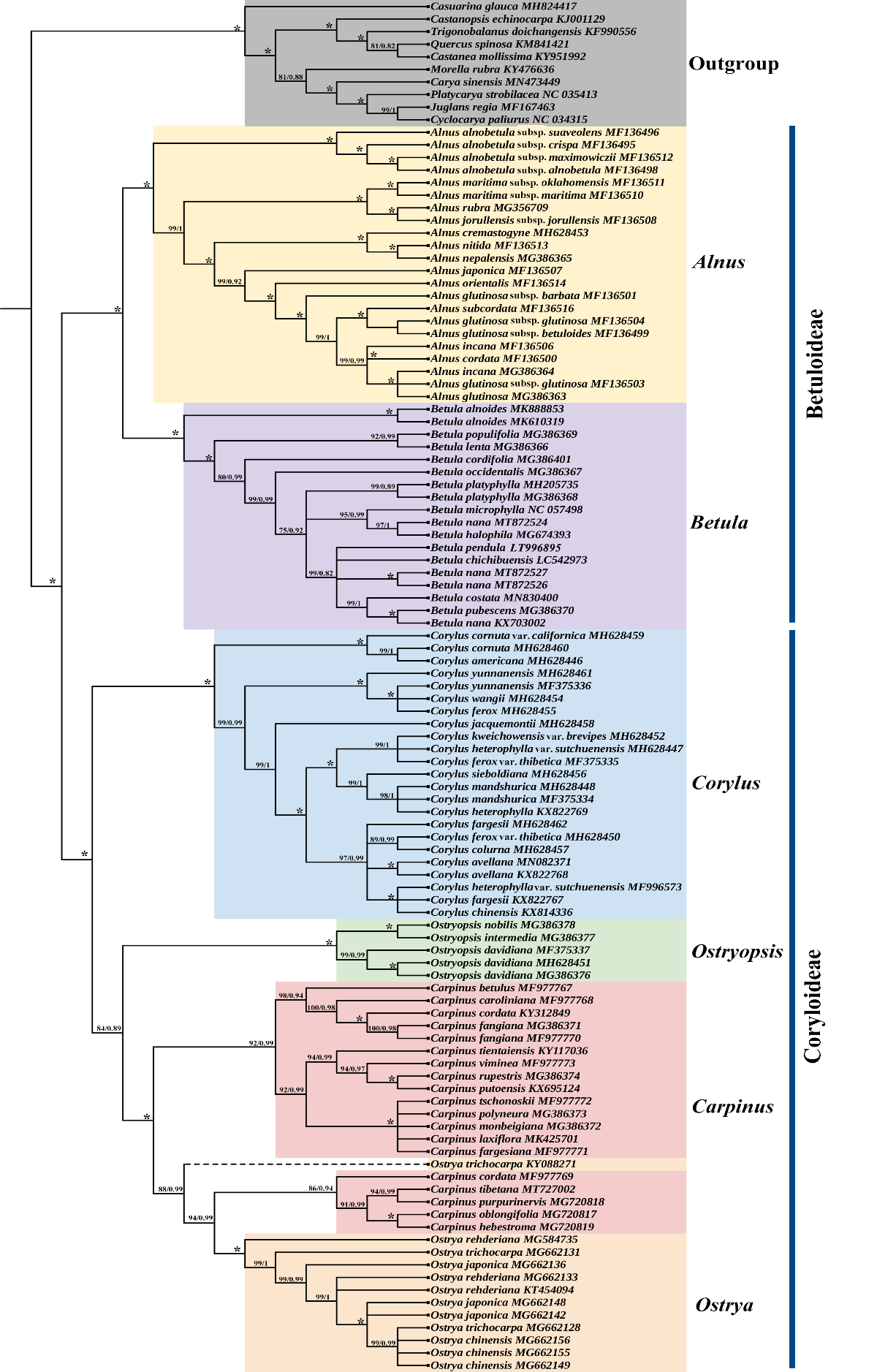


**Figure S4. Phylogeny of Betulaceae inferred from Maximum likelihood (ML) and Bayesian inference (BI) based on divergence hotspots (DH).** BS and PP values are presented on the branches. Asterisks represent 100/1.0 support values. Major genera of Betulaceae are indicated by different colors.


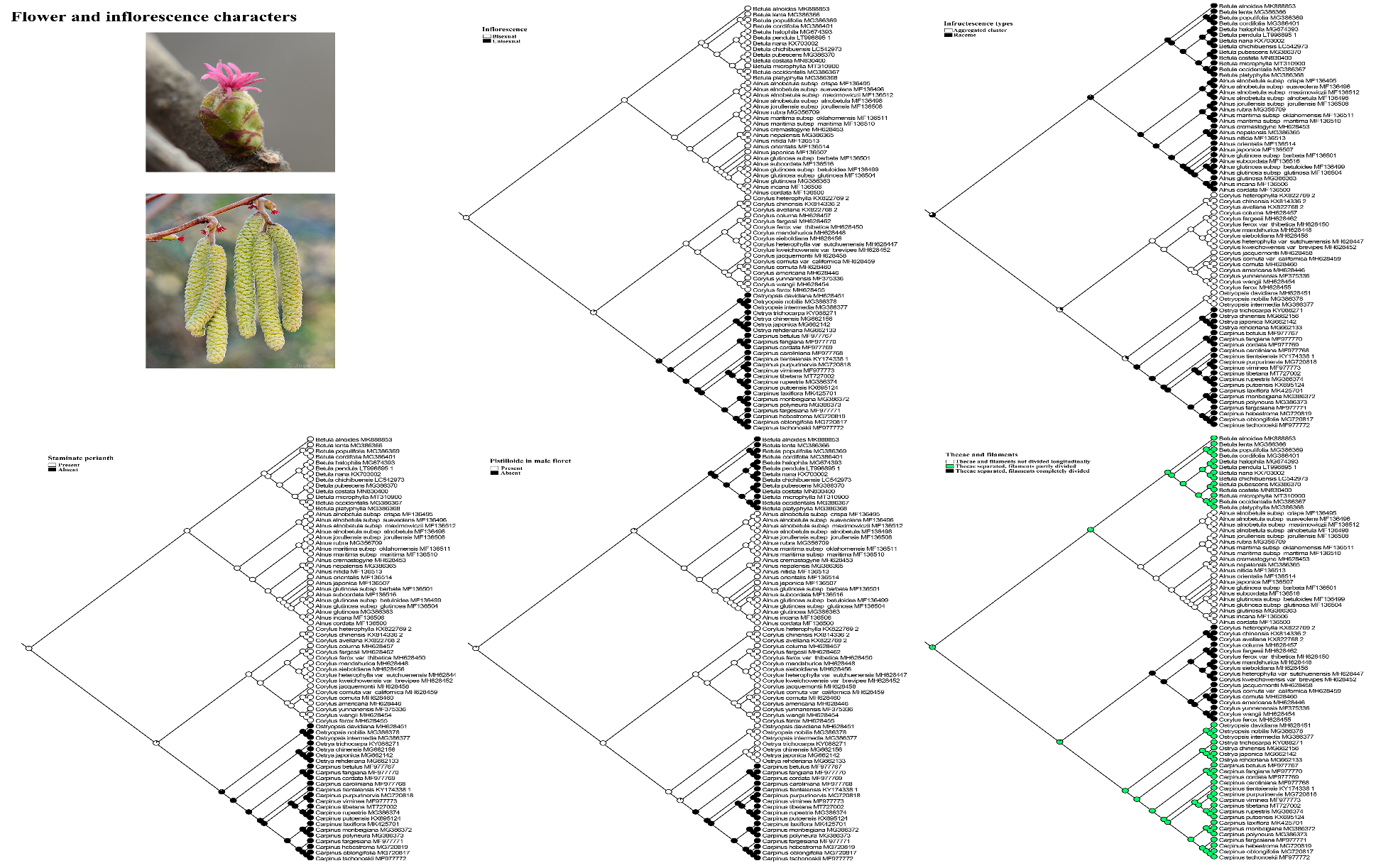


**Figure S5. Ancestral state reconstruction of five flower characters in Betulaceae.**


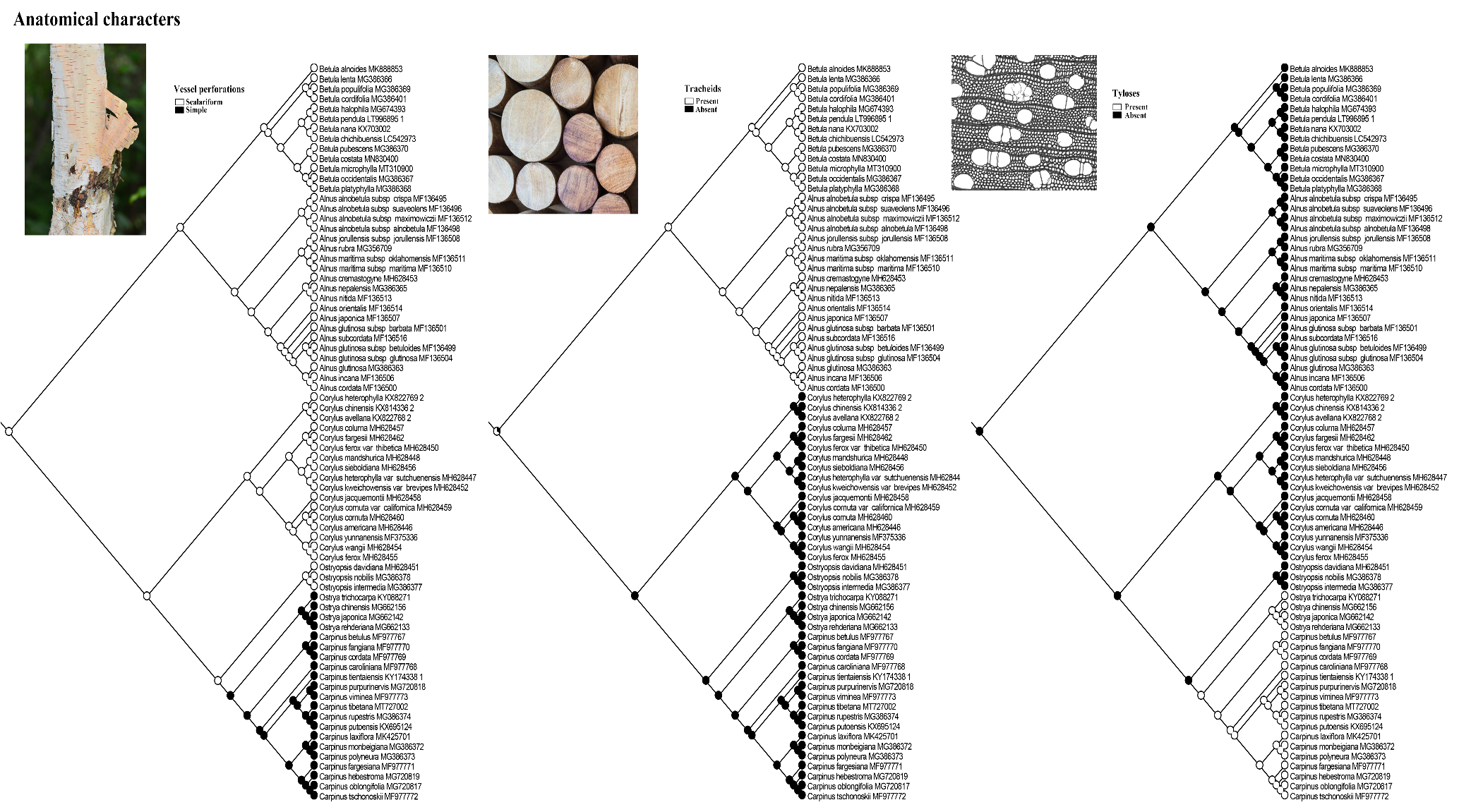


**Figure. S6**. **Ancestral state reconstruction of three anatomical characters in Betulaceae.**


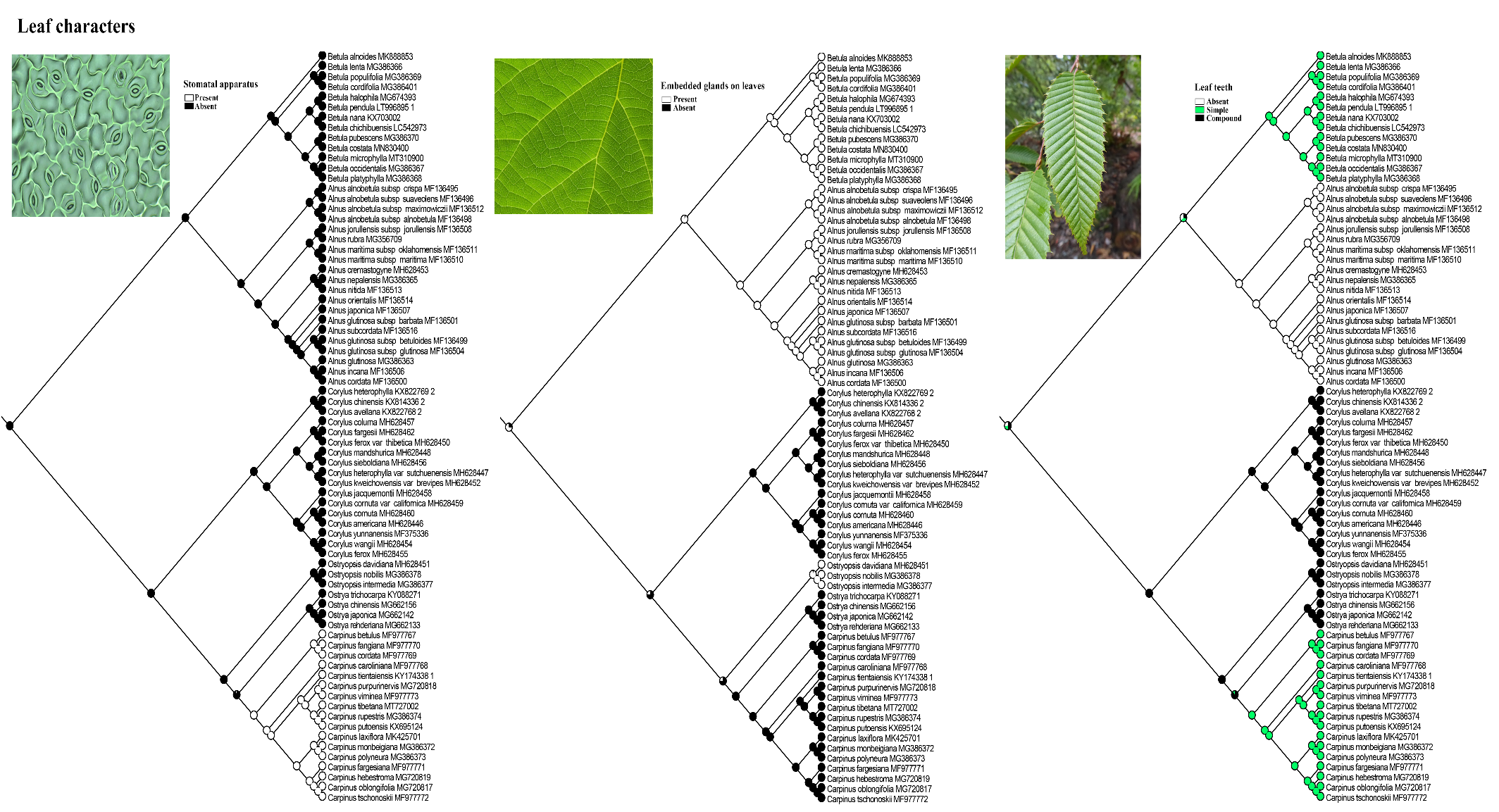


**Figure. S7**. Ancestral state reconstruction of three leaf characters in Betulaceae.
