# Supplementary material for: Plastome phylogenomics provide new perspective into the phylogeny and evolution of Betulaceae (Fagales)
Source: BMC Plant Biol. 2022 Dec 24;22:611. doi: 10.1186/s12870-022-03991-1 (PMC9789603; doi:10.1186/s12870-022-03991-1)
Supplement: Supplementary file 2 — Additional file 2: Table S1. Accession numbers and structural features of all 93 plastomes obtained from GenBank. Table S2. Summary of protein-coding genes in all Betulaceae plastomes. Table S3. Statistics of nucleotide diversity for 78 coding genes and 68 non-coding regions. Table S4. Data characteristics and best-fit models for ML and BI phylogenetic analyses. Table S5. Primers used for gap closure in this study. Table S6. Results of model test used for biogeographic inference. [file 12870_2022_3991_MOESM2_ESM.docx]

**Table S1.** Accession numbers and structural features of all 93 plastomes obtained from GenBank.

| ID | Organism | Genus | Length | LSC | SSC | IR | CDS | tRNA | rRNA | Total gene | duplicated | Single-copy | GC% |
| --- | --- | --- | --- | --- | --- | --- | --- | --- | --- | --- | --- | --- | --- |
| MF136498.1 | *Alnus alnobetula* subsp*. alnobetula* | *Alnus* | 160473 | 89256 | 19177 | 26020 | 85 | 30 | 8 | 123 | 16 | 107 | 36.4 |
| MF136495.1 | *Alnus alnobetula* subsp*. crispa* | *Alnus* | 160534 | 89211 | 19245 | 26039 | 85 | 30 | 8 | 123 | 16 | 107 | 36.4 |
| MF136512.1 | *Alnus alnobetula* subsp*. maximowiczii* | *Alnus* | 160586 | 89293 | 19231 | 26031 | 85 | 30 | 8 | 123 | 16 | 107 | 36.4 |
| MF136496.1 | *Alnus alnobetula* subsp*. suaveolens* | *Alnus* | 160570 | 89260 | 19248 | 26031 | 85 | 30 | 8 | 123 | 16 | 107 | 36.4 |
| MF136500.1 | *Alnus cordata* | *Alnus* | 160740 | 89427 | 19250 | 26030 | 85 | 30 | 8 | 123 | 16 | 107 | 36.4 |
| MH628453.1 | *Alnus cremastogyne* | *Alnus* | 160538 | 89074 | 19094 | 26185 | 84 | 37 | 8 | 129 | 17 | 112 | 36.4 |
| MG386363.1 | *Alnus glutinosa* | *Alnus* | 160770 | 89297 | 19279 | 26097 | 85 | 37 | 8 | 130 | 17 | 113 | 36.4 |
| MF136501.1 | *Alnus glutinosa* subsp*. barbata* | *Alnus* | 160916 | 89496 | 19310 | 26055 | 85 | 30 | 8 | 123 | 16 | 107 | 36.4 |
| MF136499.1 | *Alnus glutinosa* subsp*. betuloides* | *Alnus* | 160774 | 89432 | 19234 | 26054 | 85 | 30 | 8 | 123 | 16 | 107 | 36.4 |
| MF136503.1 | *Alnus glutinosa* subsp*. glutinosa* | *Alnus* | 160817 | 89430 | 19279 | 26054 | 85 | 30 | 8 | 123 | 16 | 107 | 36.4 |
| MF136504.1 | *Alnus glutinosa* subsp*. glutinosa* | *Alnus* | 160749 | 89435 | 19206 | 26054 | 85 | 30 | 8 | 123 | 16 | 107 | 36.4 |
| MF136506.1 | *Alnus incana* | *Alnus* | 160586 | 89209 | 19269 | 26054 | 85 | 30 | 8 | 123 | 16 | 107 | 36.5 |
| MG386364.1 | *Alnus incana* | *Alnus* | 161055 | 89571 | 19268 | 26108 | 85 | 37 | 8 | 130 | 17 | 113 | 36.4 |
| MF136507.1 | *Alnus japonica* | *Alnus* | 160788 | 89426 | 19258 | 26052 | 85 | 30 | 8 | 123 | 16 | 107 | 36.4 |
| MF136508.1 | *Alnus jorullensis* subsp*. jorullensis* | *Alnus* | 160617 | 89357 | 19192 | 26034 | 85 | 30 | 8 | 123 | 16 | 107 | 36.4 |
| MF136510.1 | *Alnus maritima* subsp*. maritima* | *Alnus* | 160550 | 89439 | 19047 | 26032 | 85 | 30 | 8 | 123 | 16 | 107 | 36.4 |
| MF136511.1 | *Alnus maritima* subsp*. oklahomensis* | *Alnus* | 160586 | 89510 | 19014 | 26031 | 85 | 30 | 8 | 123 | 16 | 107 | 36.4 |
| MG386365.1 | *Alnus nepalensis* | *Alnus* | 160735 | 89316 | 19313 | 26053 | 85 | 37 | 8 | 130 | 17 | 113 | 36.4 |
| MF136513.1 | *Alnus nitida* | *Alnus* | 160871 | 89530 | 18993 | 26174 | 85 | 30 | 8 | 123 | 16 | 107 | 36.4 |
| MF136514.1 | *Alnus orientalis* | *Alnus* | 160470 | 89099 | 19187 | 26092 | 85 | 30 | 8 | 123 | 16 | 107 | 36.4 |
| MG356709.1 | *Alnus rubra* | *Alnus* | 160752 | 89515 | 19169 | 26034 | 85 | 37 | 8 | 130 | 17 | 113 | 36.4 |
| MF136516.1 | *Alnus subcordata* | *Alnus* | 160702 | 89384 | 19210 | 26054 | 85 | 30 | 8 | 123 | 16 | 107 | 36.4 |
| MK610319.1 | *Betula alnoides* | *Betula* | 161022 | 89440 | 19535 | 26022 | 84 | 37 | 8 | 129 | 17 | 112 | 35.9 |
| MK888853.1 | *Betula alnoides* | *Betula* | 160990 | 89719 | 19227 | 26022 | 85 | 37 | 8 | 130 | 17 | 113 | 36 |
| LC542973.1 | *Betula chichibuensis* | *Betula* | 160791 | 89504 | 19175 | 26056 | 88 | 37 | 8 | 133 | 20 | 113 | 36 |
| MG386401.1 | *Betula cordifolia* | *Betula* | 160771 | 89581 | 19110 | 26040 | 85 | 37 | 8 | 130 | 17 | 113 | 36 |
| MN830400.1 | *Betula costata* | *Betula* | 160547 | 89385 | 19038 | 26062 | 86 | 37 | 8 | 131 | 17 | 114 | 36 |
| MG674393.1 | *Betula halophila* | *Betula* | 160648 | 89552 | 19026 | 26035 | 85 | 40 | 8 | 133 | 19 | 114 | 36 |
| MG386366.1 | *Betula lenta* | *Betula* | 160618 | 89382 | 19298 | 25969 | 85 | 37 | 8 | 130 | 17 | 113 | 36.1 |
| NC_057498.1 | *Betula microphylla* | *Betula* | 160489 | 89306 | 19045 | 26070 | 83 | 37 | 8 | 128 | 15 | 113 | 36 |
| KX703002.1 | *Betula nana* | *Betula* | 160579 | 89493 | 19018 | 26034 | 86 | 40 | 8 | 134 | 20 | 114 | 36 |
| MT872524.1 | *Betula nana* | *Betula* | 160518 | 89397 | 19009 | 26056 | 86 | 40 | 8 | 134 | 20 | 114 | 36 |
| MT872526.1 | *Betula nana* | *Betula* | 160466 | 89491 | 19001 | 25987 | 85 | 40 | 8 | 133 | 19 | 114 | 36 |
| MT872527.1 | *Betula nana* | *Betula* | 160480 | 89423 | 19001 | 26028 | 85 | 40 | 8 | 133 | 19 | 114 | 36.1 |
| MG386367.1 | *Betula occidentalis* | *Betula* | 160309 | 89513 | 18730 | 26033 | 85 | 37 | 8 | 130 | 17 | 113 | 36.1 |
| MG966529.2 | *Betula pendula* var*. carelica* | *Betula* | 160534 | 89472 | 19006 | 26028 | 83 | 37 | 8 | 128 | 14 | 114 | 36 |
| MG386368.1 | *Betula platyphylla* | *Betula* | 161349 | 90272 | 19009 | 26034 | 84 | 37 | 8 | 129 | 17 | 112 | 36 |
| MH205735.1 | *Betula platyphylla* | *Betula* | 160518 | 89397 | 19009 | 26056 | 85 | 37 | 8 | 130 | 17 | 113 | 36 |
| MG386369.1 | *Betula populifolia* | *Betula* | 160263 | 89490 | 18735 | 26014 | 85 | 37 | 8 | 130 | 17 | 113 | 36 |
| MG386370.1 | *Betula pubescens* | *Betula* | 158647 | 88568 | 18097 | 25991 | 85 | 37 | 8 | 130 | 17 | 113 | 36.4 |
| MF977767.1 | *Carpinus betulus* | *Carpinus* | 160583 | 88282 | 17167 | 27567 | 84 | 29 | 8 | 121 | 14 | 107 | 36.4 |
| MF977768.1 | *Carpinus caroliniana* | *Carpinus* | 160151 | 88555 | 18514 | 26541 | 84 | 29 | 8 | 121 | 14 | 107 | 36.4 |
| KY312849.1 | *Carpinus cordata* | *Carpinus* | 159157 | 88525 | 18557 | 26038 | 84 | 36 | 8 | 128 | 17 | 112 | 36.4 |
| MF977769.1 | *Carpinus cordata* | *Carpinus* | 158865 | 87939 | 18800 | 26063 | 85 | 29 | 8 | 122 | 14 | 107 | 36.5 |
| MF977770.1 | *Carpinus fangiana* | *Carpinus* | 159222 | 88168 | 18537 | 26041 | 84 | 29 | 8 | 121 | 14 | 107 | 36.5 |
| MG386371.1 | *Carpinus fangiana* | *Carpinus* | 158787 | 88280 | 18826 | 26058 | 85 | 37 | 8 | 130 | 17 | 113 | 36.5 |
| MF977771.1 | *Carpinus fargesiana* | *Carpinus* | 159484 | 88520 | 18816 | 26074 | 84 | 29 | 8 | 121 | 14 | 107 | 36.4 |
| MG720819.1 | *Carpinus hebestroma* | *Carpinus* | 159231 | 88168 | 18873 | 26095 | 85 | 29 | 8 | 122 | 15 | 107 | 36.5 |
| MK425701.1 | *Carpinus laxiflora* | *Carpinus* | 159255 | 88359 | 18764 | 26066 | 87 | 37 | 8 | 132 | 19 | 113 | 36.5 |
| MG386372.1 | *Carpinus monbeigiana* | *Carpinus* | 159450 | 88553 | 18775 | 26061 | 85 | 37 | 8 | 130 | 17 | 113 | 36.4 |
| MG720817.1 | *Carpinus oblongifolia* | *Carpinus* | 159086 | 88190 | 18764 | 26066 | 85 | 29 | 8 | 122 | 15 | 107 | 36.5 |
| MG386373.1 | *Carpinus polyneura* | *Carpinus* | 159400 | 88516 | 18764 | 26060 | 85 | 37 | 8 | 130 | 17 | 113 | 36.4 |
| MG720818.1 | *Carpinus purpurinervis* | *Carpinus* | 159145 | 88187 | 18800 | 26079 | 85 | 29 | 8 | 122 | 15 | 107 | 36.5 |
| KX695124.1 | *Carpinus putoensis* | *Carpinus* | 159673 | 89020 | 18567 | 26043 | 84 | 36 | 8 | 128 | 16 | 112 | 36.5 |
| MG386374.1 | *Carpinus rupestris* | *Carpinus* | 158888 | 87972 | 18780 | 26068 | 85 | 37 | 8 | 130 | 17 | 113 | 36.5 |
| MT727002.1 | *Carpinus tibetana* | *Carpinus* | 158762 | 87825 | 18797 | 26070 | 85 | 35 | 8 | 128 | 16 | 112 | 36.5 |
| KY117036.1 | *Carpinus tientaiensis* | *Carpinus* | 160104 | 89446 | 18598 | 26030 | 85 | 36 | 8 | 129 | 16 | 113 | 36.4 |
| MF977772.1 | *Carpinus tschonoskii* | *Carpinus* | 159505 | 88617 | 18760 | 26064 | 84 | 29 | 8 | 121 | 14 | 107 | 36.4 |
| MF977773.1 | *Carpinus viminea* | *Carpinus* | 158681 | 87808 | 18807 | 26033 | 84 | 29 | 8 | 121 | 14 | 107 | 36.5 |
| MH628448.1 | *Corylus mandshurica* | *Corylus* | 159828 | 88850 | 18782 | 26098 | 84 | 36 | 8 | 128 | 17 | 111 | 36.4 |
| MH628456.1 | *Corylus sieboldiana* | *Corylus* | 159870 | 88865 | 18795 | 26105 | 84 | 36 | 8 | 128 | 17 | 111 | 36.4 |
| MH628454.1 | *Corylus wangii* | *Corylus* | 159825 | 88743 | 18870 | 26106 | 91 | 37 | 8 | 136 | 17 | 113 | 36.5 |
| MH628461.1 | *Corylus yunnanensis* | *Corylus* | 160085 | 89043 | 18840 | 26101 | 84 | 36 | 8 | 128 | 16 | 111 | 36.4 |
| MH628447.1 | *Corylus heterophylla* var*. sutchuenensis* | *Corylus* | 159887 | 88886 | 18805 | 26098 | 84 | 36 | 8 | 128 | 17 | 111 | 36.4 |
| MH628458.1 | *Corylus jacquemontii* | *Corylus* | 159862 | 88846 | 18818 | 26099 | 84 | 36 | 8 | 128 | 17 | 111 | 36.4 |
| MH628452.1 | *Corylus kweichowensis* var*. brevipes* | *Corylus* | 159822 | 88850 | 18776 | 26098 | 84 | 36 | 8 | 128 | 17 | 111 | 36.4 |
| MH628446.1 | *Corylus americana* | *Corylus* | 159912 | 88895 | 18813 | 26102 | 83 | 36 | 8 | 127 | 17 | 111 | 36.4 |
| MH628457.1 | *Corylus colurna* | *Corylus* | 159842 | 88873 | 18773 | 26098 | 84 | 36 | 8 | 128 | 17 | 111 | 36.4 |
| MH628460.1 | *Corylus cornuta* | *Corylus* | 159857 | 88877 | 18796 | 26092 | 84 | 36 | 8 | 128 | 17 | 111 | 36.4 |
| MH628459.1 | *Corylus cornuta* var*. californica* | *Corylus* | 159915 | 88867 | 18844 | 26102 | 84 | 36 | 8 | 128 | 17 | 111 | 36.4 |
| MH628462.1 | *Corylus fargesii* | *Corylus* | 159838 | 88879 | 18777 | 26091 | 83 | 36 | 8 | 127 | 16 | 111 | 36.4 |
| MH628455.1 | *Corylus ferox* | *Corylus* | 159825 | 88742 | 18869 | 26107 | 84 | 36 | 8 | 128 | 17 | 111 | 36.5 |
| MH628450.1 | *Corylus ferox* var*. thibetica* | *Corylus* | 160358 | 88357 | 18769 | 26616 | 84 | 36 | 8 | 128 | 17 | 111 | 36.4 |
| KX822768.2 | *Corylus avellana* | *Corylus* | 160160 | 88160 | 18766 | 26617 | 85 | 36 | 8 | 129 | 17 | 111 | 36.5 |
| MN082371.1 | *Corylus avellana* | *Corylus* | 161667 | 90197 | 18732 | 26369 | 84 | 34 | 8 | 126 | 23 | 109 | 36.4 |
| KX814336.2 | *Corylus chinensis* | *Corylus* | 160320 | 88340 | 18778 | 26601 | 84 | 36 | 8 | 128 | 17 | 111 | 36.4 |
| KX822767.2 | *Corylus fargesii* | *Corylus* | 160322 | 88341 | 18777 | 26602 | 84 | 36 | 8 | 128 | 17 | 111 | 36.4 |
| MF375335.1 | *Corylus ferox* var*. thibetica* | *Corylus* | 159894 | 88898 | 18800 | 26098 | 83 | 36 | 8 | 127 | 16 | 111 | 36.4 |
| KX822769.2 | *Corylus heterophylla* | *Corylus* | 160350 | 88434 | 18782 | 26567 | 84 | 36 | 8 | 128 | 17 | 111 | 36.4 |
| MF996573.1 | *Corylus heterophylla* var*. sutchuenensis* | *Corylus* | 161127 | 89119 | 18778 | 26615 | 84 | 37 | 8 | 129 | 18 | 112 | 36.4 |
| MF375334.1 | *Corylus mandshurica* | *Corylus* | 159833 | 88853 | 18784 | 26098 | 83 | 36 | 8 | 127 | 16 | 111 | 36.4 |
| MF375336.1 | *Corylus yunnanensis* | *Corylus* | 159769 | 88726 | 18829 | 26107 | 83 | 36 | 8 | 127 | 16 | 111 | 36.5 |
| MG662149.1 | *Ostrya chinensis* | *Ostrya* | 159293 | 88203 | 18974 | 26058 | 85 | 29 | 8 | 122 | 15 | 107 | 36.5 |
| MG662155.1 | *Ostrya chinensis* | *Ostrya* | 159154 | 88138 | 18906 | 26055 | 85 | 29 | 8 | 122 | 15 | 107 | 36.5 |
| MG662156.1 | *Ostrya chinensis* | *Ostrya* | 159265 | 88229 | 18912 | 26062 | 85 | 29 | 8 | 122 | 15 | 107 | 36.5 |
| MG662136.1 | *Ostrya japonica* | *Ostrya* | 159236 | 88169 | 18937 | 26065 | 85 | 29 | 8 | 122 | 15 | 107 | 36.5 |
| MG662142.1 | *Ostrya japonica* | *Ostrya* | 159245 | 88185 | 18944 | 26058 | 85 | 29 | 8 | 122 | 15 | 107 | 36.5 |
| MG662148.1 | *Ostrya japonica* | *Ostrya* | 159245 | 88183 | 18944 | 26059 | 85 | 29 | 8 | 122 | 15 | 107 | 36.5 |
| KT454094.1 | *Ostrya rehderiana* | *Ostrya* | 159347 | 88177 | 18908 | 26131 | 85 | 31 | 8 | 124 | 16 | 108 | 36.5 |
| MG584735.1 | *Ostrya rehderiana* | *Ostrya* | 159108 | 88458 | 18792 | 25929 | 85 | 30 | 8 | 123 | 16 | 107 | 36.5 |
| MG662133.1 | *Ostrya rehderiana* | *Ostrya* | 159241 | 88180 | 18943 | 26059 | 85 | 29 | 8 | 122 | 15 | 107 | 36.5 |
| KY088271.1 | *Ostrya trichocarpa* | *Ostrya* | 159122 | 88154 | 18758 | 26105 | 85 | 29 | 8 | 122 | 15 | 107 | 36.4 |
| MG662128.1 | *Ostrya trichocarpa* | *Ostrya* | 159300 | 88219 | 18959 | 26061 | 85 | 29 | 8 | 122 | 15 | 107 | 36.5 |
| MG662131.1 | *Ostrya trichocarpa* | *Ostrya* | 159204 | 88019 | 18859 | 26163 | 84 | 29 | 8 | 121 | 14 | 107 | 36.5 |
| MF375337.1 | *Ostryopsis davidiana* | *Ostryopsis* | 159280 | 88569 | 18583 | 26064 | 83 | 36 | 8 | 127 | 16 | 111 | 36.4 |
| MG386376.1 | *Ostryopsis davidiana* | *Ostryopsis* | 159461 | 88819 | 18546 | 26048 | 84 | 37 | 8 | 129 | 17 | 112 | 36.5 |
| MH628451.1 | *Ostryopsis davidiana* | *Ostryopsis* | 159286 | 88568 | 18588 | 26065 | 85 | 37 | 8 | 130 | 17 | 113 | 36.5 |
| MG386377.1 | *Ostryopsis intermedia* | *Ostryopsis* | 159213 | 88450 | 18619 | 26072 | 85 | 37 | 8 | 130 | 17 | 113 | 36.5 |
| MG386378.1 | *Ostryopsis nobilis* | *Ostryopsis* | 160908 | 90144 | 18620 | 26072 | 85 | 37 | 8 | 130 | 17 | 113 | 36.5 |
| KY951992 | *Castanea mollissima* | outgroup | 160869 |  |  |  |  |  |  |  |  |  |  |
| KJ001129 | *Castanopsis echinocarpa* | outgroup | 160647 |  |  |  |  |  |  |  |  |  |  |
| MF167463 | *Juglans regia* | outgroup | 160370 |  |  |  |  |  |  |  |  |  |  |
| KY476636 | *Morella rubra isolate FJZS* | outgroup | 159568 |  |  |  |  |  |  |  |  |  |  |
| KM841421 | *Quercus spinosa* | outgroup | 160825 |  |  |  |  |  |  |  |  |  |  |
| KF990556 | *Trigonobalanus doichangensis* | outgroup | 159938 |  |  |  |  |  |  |  |  |  |  |
| MH824417 | *Casuarina?glauca* | outgroup | 155669 |  |  |  |  |  |  |  |  |  |  |
| NC_035413.1 | *Platycarya strobilacea* | outgroup | 160994 |  |  |  |  |  |  |  |  |  |  |
| MN473449 | *Annamocarya sinensis* | outgroup | 160065 |  |  |  |  |  |  |  |  |  |  |
| NC_034315.1 | *Cyclocarya paliurus* | outgroup | 160562 |  |  |  |  |  |  |  |  |  |  |

**Table S2.** Summary of protein-coding genes in all Betulaceae plastomes

| ID | Organism | Number of coding genes | Lost genes | Shared genes |
| --- | --- | --- | --- | --- |
| MF136498.1 | *Alnus alnobetula* subsp. *alnobetula* | 79 | *infA* | *accD*  *atpA*  *atpB*  *atpE*  *atpF*  *atpH*  *atpI*  *ccsA*  *cemA*  *clpP*  *matK*  *ndhA*  *ndhB*  *ndhC*  *ndhD*  *ndhE*  *ndhF*  *ndhG*  *ndhH*  *ndhI*  *ndhJ*  *ndhK*  *petA*  *petB*  *petD*  *petG*  *petL*  *petN*  *psaA*  *psaB*  *psaC*  *psaI*  *psaJ*  *psbA*  *psbB*  *psbC*  *psbD*  *psbE*  *psbF*  *psbH*  *psbI*  *psbJ*  *psbK*  *psbL*  *psbM*  *psbN*  *psbT*  *psbZ*  *rbcL*  *rpl2*  *rpl14*  *rpl16*  *rpl20*  *rpl22*  *rpl23*  *rpl32*  *rpl33*  *rpl36*  *rpoA*  *rpoB*  *rpoC1*  *rpoC2*  *rps2*  *rps3*  *rps4*  *rps7*  *rps8*  *rps11*  *rps12*  *rps14*  *rps15*  *rps16*  *rps18*  *rps19*  *ycf1*  *ycf2*  *ycf3*  *ycf4* |
| MF136495.1 | *Alnus alnobetula* subsp. *crispa* | 79 | *infA* |  |
| MF136512.1 | *Alnus alnobetula* subsp. *maximowiczii* | 79 | *infA* |  |
| MF136496.1 | *Alnus alnobetula* subsp. *suaveolens* | 79 | *infA* |  |
| MF136500.1 | *Alnus cordata* | 79 | *infA* |  |
| MH628453.1 | *Alnus cremastogyne* | 78 | *infA, ycf15* |  |
| MG386363.1 | *Alnus glutinosa* | 79 | *infA* |  |
| MF136501.1 | *Alnus glutinosa* subsp. *barbata* | 79 | *infA* |  |
| MF136499.1 | *Alnus glutinosa* subsp. *betuloides* | 79 | *infA* |  |
| MF136503.1 | *Alnus glutinosa* subsp. *glutinosa* | 79 | *infA* |  |
| MF136504.1 | *Alnus glutinosa* subsp*. glutinosa* | 79 | *infA* |  |
| MF136506.1 | *Alnus incana* | 79 | *infA* |  |
| MG386364.1 | *Alnus incana* | 79 | *infA* |  |
| MF136507.1 | *Alnus japonica* | 79 | *infA* |  |
| MF136508.1 | *Alnus jorullensis* subsp*. jorullensis* | 79 | *infA* |  |
| MF136510.1 | *Alnus maritima* subsp*. maritima* | 79 | *infA* |  |
| MF136511.1 | *Alnus maritima* subsp*. oklahomensis* | 79 | *infA* |  |
| MG386365.1 | *Alnus nepalensis* | 79 | *infA* |  |
| MF136513.1 | *Alnus nitida* | 79 | *infA* |  |
| MF136514.1 | *Alnus orientalis* | 79 | *infA* |  |
| MG356709.1 | *Alnus rubra* | 79 | *infA* |  |
| MF136516.1 | *Alnus subcordata* | 79 | *infA* |  |
| MK888853.1 | *Betula alnoides* | 78 | *infA, ycf15* |  |
| MK610319.1 | *Betula alnoides* | 79 | *infA* |  |
| LC542973.1 | *Betula chichibuensis* | 80 | *-* |  |
| MG386401.1 | *Betula cordifolia* | 79 | *infA* |  |
| MN830400.1 | *Betula costata* | 80 | *-* |  |
| MG674393.1 | *Betula halophila* | 79 | *infA* |  |
| MG386366.1 | *Betula lenta* | 79 | *infA* |  |
| NC_057498.1 | *Betula microphylla* | 79 | *infA* |  |
| MT872524.1 | *Betula nana* | 79 | *infA* |  |
| KX703002.1 | *Betula nana* | 79 | *infA* |  |
| MT872526.1 | *Betula nana* | 79 | *infA* |  |
| MT872527.1 | *Betula nana* | 79 | *infA* |  |
| MG386367.1 | *Betula occidentalis* | 79 | *infA* |  |
| MG966529.2 | *Betula pendula* var*. carelica* | 78 | *infA, ycf15* |  |
| MH205735.1 | *Betula platyphylla* | 78 | *infA, ycf15* |  |
| MG386368.1 | *Betula platyphylla* | 79 | *infA* |  |
| MG386369.1 | *Betula populifolia* | 79 | *infA* |  |
| MG386370.1 | *Betula pubescens* | 79 | *infA* |  |
| MF977767.1 | *Carpinus betulus* | 79 | *infA* |  |
| MF977768.1 | *Carpinus caroliniana* | 79 | *infA* |  |
| MF977769.1 | *Carpinus cordata* | 79 | *infA* |  |
| KY312849.1 | *Carpinus cordata* | 79 | *infA* |  |
| MF977770.1 | *Carpinus fangiana* | 79 | *infA* |  |
| MG386371.1 | *Carpinus fangiana* | 79 | *infA* |  |
| MF977771.1 | *Carpinus fargesiana* | 79 | *infA* |  |
| MG720819.1 | *Carpinus hebestroma* | 79 | *infA* |  |
| MK425701.1 | *Carpinus laxiflora* | 80 |  |  |
| MG386372.1 | *Carpinus monbeigiana* | 79 | *infA* |  |
| MG720817.1 | *Carpinus oblongifolia* | 79 | *infA* |  |
| MG386373.1 | *Carpinus polyneura* | 79 | *infA* |  |
| MG720818.1 | *Carpinus purpurinervis* | 79 | *infA* |  |
| KX695124.1 | *Carpinus putoensis* | 78 | *infA, ycf15* |  |
| MG386374.1 | *Carpinus rupestris* | 79 | *infA* |  |
| MT727002.1 | *Carpinus tibetana* | 79 | *infA* |  |
| KY117036.1 | *Carpinus tientaiensis* | 79 | *infA* |  |
| MF977772.1 | *Carpinus tschonoskii* | 79 | *infA* |  |
| MF977773.1 | *Carpinus viminea* | 79 | *infA* |  |
| MH628446.1 | *Corylus americana* | 78 | *infA, ycf15* |  |
| KX822768.2 | *Corylus avellana* | 78 | *infA, ycf15* |  |
| MN082371.1 | *Corylus avellana* | 79 | *infA* |  |
| KX814336.2 | *Corylus chinensis* | 78 | *infA, ycf15* |  |
| MH628457.1 | *Corylus colurna* | 78 | *infA, ycf15* |  |
| MH628460.1 | *Corylus cornuta* | 78 | *infA, ycf15* |  |
| MH628459.1 | *Corylus cornuta* var. *californica* | 78 | *infA, ycf15* |  |
| MH628462.1 | *Corylus fargesii* | 78 | *infA, ycf15* |  |
| KX822767.2 | *Corylus fargesii* | 78 | *infA, ycf15* |  |
| MH628455.1 | *Corylus ferox* | 78 | *infA, ycf15* |  |
| MH628450.1 | *Corylus ferox* var. *thibetica* | 78 | *infA, ycf15* |  |
| MF375335.1 | *Corylus ferox* var*. thibetica* | 78 | *infA, ycf15* |  |
| KX822769.2 | *Corylus heterophylla* | 78 | *infA, ycf15* |  |
| MH628447.1 | *Corylus heterophylla* var*. sutchuenensis* | 78 | *infA, ycf15* |  |
| MF996573.1 | *Corylus heterophylla* var. *sutchuenensis* | 78 | *infA, ycf15* |  |
| MH628458.1 | *Corylus jacquemontii* | 78 | *infA, ycf15* |  |
| MH628452.1 | *Corylus kweichowensis* var*. brevipes* | 78 | *infA, ycf15* |  |
| MH628448.1 | *Corylus mandshurica* | 78 | *infA, ycf15* |  |
| MF375334.1 | *Corylus mandshurica* | 78 | *infA, ycf15* |  |
| MH628456.1 | *Corylus sieboldiana* | 78 | *infA, ycf15* |  |
| MH628454.1 | *Corylus wangii* | 78 | *infA, ycf15* |  |
| MH628461.1 | *Corylus yunnanensis* | 78 | *infA, ycf15* |  |
| MF375336.1 | *Corylus yunnanensis* | 78 | *infA, ycf15* |  |
| MG662149.1 | *Ostrya chinensis* | 79 | *infA* |  |
| MG662155.1 | *Ostrya chinensis* | 79 | *infA* |  |
| MG662156.1 | *Ostrya chinensis* | 79 | *infA* |  |
| MG662136.1 | *Ostrya japonica* | 79 | *infA* |  |
| MG662142.1 | *Ostrya japonica* | 79 | *infA* |  |
| MG662148.1 | *Ostrya japonica* | 79 | *infA* |  |
| KT454094.1 | *Ostrya rehderiana* | 79 | *infA* |  |
| MG584735.1 | *Ostrya rehderiana* | 79 | *infA* |  |
| MG662133.1 | *Ostrya rehderiana* | 79 | *infA* |  |
| KY088271.1 | *Ostrya trichocarpa* | 79 | *infA* |  |
| MG662128.1 | *Ostrya trichocarpa* | 79 | *infA* |  |
| MG662131.1 | *Ostrya trichocarpa* | 79 | *infA* |  |
| MF375337.1 | *Ostryopsis davidiana* | 78 | *infA, ycf15* |  |
| MH628451.1 | *Ostryopsis davidiana* | 78 | *infA, ycf15* |  |
| MG386376.1 | *Ostryopsis davidiana* | 79 | *infA, ycf15* |  |
| MG386377.1 | *Ostryopsis intermedia* | 79 | *infA* |  |
| MG386378.1 | *Ostryopsis nobilis* | 79 | *infA* |  |

**Table S3.** Statistics of nucleotide diversity for 78 coding genes and 68 non-coding regions.

| Protein-coding genes | *P*_i_ | Length | Non-coding regions | *P*_i_ | Length |
| --- | --- | --- | --- | --- | --- |
| *accD* | 0.01019 | 1620 | *accD_psaI* | 0.02813 | 957 |
| *atpA* | 0.00685 | 1521 | *atpB_rbcL* | 0.01534 | 758 |
| *atpB* | 0.00549 | 1473 | *atpF_atpH* | 0.02533 | 485 |
| *atpE* | 0.0054 | 399 | *atpH_atpI* | 0.02613 | 1266 |
| *atpF* | 0.01225 | 1104 | *atpI_rps2* | 0.01171 | 210 |
| *atpH* | 0.00608 | 243 | *ccsA_ndhD* | 0.02879 | 297 |
| *atpI* | 0.00682 | 744 | *cemA_petA* | 0.01249 | 212 |
| *ccsA* | 0.00882 | 954 | *clpP_psbB* | 0.014 | 465 |
| *cemA* | 0.01983 | 687 | *matK_rps16* | 0.02658 | 1494 |
| *clpP* | 0.01011 | 594 | *ndhB_rps7* | 0.00498 | 317 |
| *matK* | 0.0092 | 1512 | *ndhC_trnM-CAT* | 0.01948 | 1249 |
| *ndhA* | 0.01094 | 1092 | *ndhC_trnV-UAC* | 0.04909 | 837 |
| *ndhB* | 0.00062 | 1536 | *ndhE_ndhG* | 0.02973 | 226 |
| *ndhC* | 0.00763 | 360 | *ndhF_rpl32* | 0.02788 | 910 |
| *ndhD* | 0.01301 | 1527 | *ndhG_ndhI* | 0.02441 | 391 |
| *ndhE* | 0.01107 | 303 | *petA_psbJ* | 0.02074 | 1069 |
| *ndhF* | 0.01588 | 2266 | *petB_petD* | 0.01312 | 209 |
| *ndhG* | 0.00808 | 528 | *petD_rpoA* | 0.02425 | 222 |
| *ndhH* | 0.00896 | 1179 | *petN_psbM* | 0.02047 | 983 |
| *ndhI* | 0.01227 | 528 | *psaA_ycf3* | 0.01401 | 776 |
| *ndhJ* | 0.01033 | 474 | *psaC_ndhE* | 0.02538 | 274 |
| *ndhK* | 0.01187 | 735 | *psaI_ycf4* | 0.02385 | 410 |
| *petA* | 0.01269 | 964 | *psaJ_rpl33* | 0.03112 | 473 |
| *petB* | 0.00995 | 645 | *psbA_matK* | 0.02609 | 591 |
| *petD* | 0.00797 | 522 | *psbC_trnS-TGA* | 0.02991 | 250 |
| *petG* | 0.00036 | 111 | *psbE_petL* | 0.02337 | 1289 |
| *petL* | 0.01253 | 93 | *psbK_psbI* | 0.02811 | 411 |
| *petN* | 0.00345 | 96 | *psbM_trnD-GTC* | 0.01974 | 1150 |
| *psaA* | 0.00523 | 2250 | *psbZ_trnG-GCC* | 0.01825 | 282 |
| *psaB* | 0.0066 | 2202 | *rbcL_accD* | 0.024 | 708 |
| *psaC* | 0.00332 | 243 | *rpl20_rps12* | 0.02148 | 809 |
| *psaI* | 0.04403 | 111 | *rpl32_trnL-TAG* | 0.03088 | 823 |
| *psaJ* | 0.02227 | 126 | *rpl36_rps8* | 0.02582 | 459 |
| *psbA* | 0.00352 | 1059 | *rpoB_trnC-GCA* | 0.01759 | 1141 |
| *psbB* | 0.00676 | 1524 | *rps2_rpoC2* | 0.0293 | 219 |
| *psbC* | 0.00478 | 1461 | *rps4_trnT-TGT* | 0.01046 | 333 |
| *psbD* | 0.00519 | 1059 | *rps7_trnV-GAC* | 0.00238 | 2539 |
| *psbE* | 0.00505 | 249 | *rps15_ycf1* | 0.0211 | 295 |
| *psbF* | 0.00791 | 117 | *rps16_trnQ-TTG* | 0.02537 | 1337 |
| *psbH* | 0.00161 | 228 | *rps18_rpl20* | 0.02698 | 267 |
| *psbI* | 0.00378 | 156 | *rrn16S_rrn23S* | 0.00386 | 2421 |
| *psbJ* | 0.0025 | 120 | *rrn5S_trnR-ACG* | 0.00118 | 255 |
| *psbK* | 0.01924 | 183 | *trnC-GCA_petN* | 0.01694 | 954 |
| *psbL* | 0.00997 | 114 | *trnD-GTC_trnY-GTA* | 0.02011 | 469 |
| *psbM* | 0.00469 | 102 | *trnE-TTC_trnT-GGT* | 0.08544 | 751 |
| *psbN* | 0.01166 | 129 | *trnF-GAA_ndhJ* | 0.02112 | 727 |
| *psbT* | 0.00434 | 114 | *trnG-GCC_trnfM-CAT* | 0.00787 | 205 |
| *psbZ* | 0.0032 | 186 | *trnH-GTG_psbA* | 0.04257 | 409 |
| *rbcL* | 0.0088 | 1446 | *trnL-CAA_ndhB* | 0.00231 | 576 |
| *rpl2* | 0.01194 | 366 | *trnM-CAT_atpE* | 0.02578 | 206 |
| *rpl14* | 0.00887 | 408 | *trnN-GTT_ndhF* | 0.00252 | 1723 |
| *rpl16* | 0.00292 | 861 | *trnP-TGG_psaJ* | 0.03028 | 358 |
| *rpl20* | 0.01353 | 351 | *trnQ-TTG_psbK* | 0.01384 | 437 |
| *rpl22* | 0.02485 | 531 | *trnR-ACG_trnN-GTT* | 0.00621 | 605 |
| *rpl23* | 0.01358 | 291 | *trnR-TCT_atpA* | 0.0364 | 209 |
| *rpl32* | 0.00927 | 184 | *trnS-GCT_trnR-TCT* | 0.02035 | 1736 |
| *rpl33* | 0.00906 | 219 | *trnS-GGA_rps4* | 0.01681 | 309 |
| *rpl36* | 0.00902 | 129 | *trnS-TGA_psbZ* | 0.01316 | 380 |
| *rpoA* | 0.00888 | 993 | *trnT-GGT_psbD* | 0.11003 | 1308 |
| *rpoB* | 0.00649 | 3210 | *trnT-TGT_trnF-GAA* | 0.02489 | 1740 |
| *rpoC1* | 0.00577 | 2076 | *trnT-TGT_trnL-UAA* | 0.02146 | 927 |
| *rpoC2* | 0.00903 | 4206 | *trnV-GAC_rrn16S* | 0.00068 | 228 |
| *rps2* | 0.01128 | 414 | *ycf15_trnL-CAA* | 0.0005 | 462 |
| *rps3* | 0.00278 | 379 | *ycf3_trnS-GGA* | 0.02464 | 408 |
| *rps4* | 0.00689 | 300 | *ycf4_cemA* | 0.03716 | 412 |
| *rps7* | 0.01323 | 279 |  |  |  |
| *rps8* | 0.00648 | 261 |  |  |  |
| *rps11* | 0.00451 | 303 |  |  |  |
| *rps12* | 0.01124 | 276 |  |  |  |
| *rps14* | 0.00726 | 723 |  |  |  |
| *rps15* | 0.00706 | 657 |  |  |  |
| *rps16* | 0.00464 | 603 |  |  |  |
| *rps18* | 0.00065 | 465 |  |  |  |
| *rps19* | 0.01303 | 402 |  |  |  |
| *ycf1* | 0.0253 | 5934 |  |  |  |
| *ycf2* | 0.00237 | 6897 |  |  |  |
| *ycf3* | 0.00236 | 507 |  |  |  |
| *ycf4* | 0.00785 | 552 |  |  |  |

**Table S4.** Data characteristics and best-fit models for ML and BI phylogenetic analyses

| Datasets | Abbr. | Aligned length | Parsimony sites | ML model | BI model |
| --- | --- | --- | --- | --- | --- |
| Protein-coding sequences | CDS | 67,157 bp | 2529 (3.77%) | GTR+F+R3 | GTR+F+G4 |
| Non-coding sequences | CNS | 45,608 bp | 4263 (9.35%) | TVM+F+R3 | GTR+F+G4+I |
| Whole plastomes | WP | 155,530 bp | 16310 (10.49%) | TVM+F+R4 | GTR+F+G+I |
| DNA barcodes | DH | 10,592 bp | 2507 (23.67%) | TVM+F+R3 | GTR+F+G4 |

**Table S5.** Primers used for gap closure in this study.

| Primer | Direction | Sequence | Tm (℃) |
| --- | --- | --- | --- |
| 1 | F | CCCTGTAAGGCTTGTTG | 54.5 |
|  | R | ATAGGTCGTCGATTCGGC |  |
| 2 | F | AACCCGACTTCCCAAGGA | 56.2 |
|  | R | CGTTGATCTAGTCGCCAC |  |
| 3 | F | CCGTCTCGGTATCTGGGTAA | 57.5 |
|  | R | GCAAAGAACTAAGTGGGCG |  |
| 4 | F | TGCGAGCTACCAAACTGC | 55.4 |
|  | R | TCTGTCGTTTGTTGTCCCAC |  |
| 5 | F | CCGACGGTTCACAAGCAG | 57 |
|  | R | GTCGTTCTATGGGATCGC |  |
| 6 | F | CTGAGCGGCAGCTACAAA | 55.6 |
|  | R | CCTTGCAGCTCGTATTGT |  |

The PCR program began with 4-min initial denaturing at 94 ºС followed by 35 cycles of 1-min denaturation at 94 ºС, 1-min annealing at abovementioned Tm, and 1.5-min extension at 72 ºС, a final extension was run for 5 min at 72 ºС.

**Table S6.** Results of model test used for biogeographic inference.

| Models | LnL | Numparams | d | e | j | AICc | AICc_wt |
| --- | --- | --- | --- | --- | --- | --- | --- |
| BAYAREALIKE | -251.3 | 2 | 0.037 | 0.36 | 0 | 506.8 | 0.55 |
| BAYAREALIKE+J | -251.3 | 3 | 0.036 | 0.35 | 1.0e-05 | 508.9 | 0.19 |
| DEC | -252.4 | 2 | 0.034 | 0.29 | 0 | 508.9 | 0.18 |
| DEC+J | -252.6 | 3 | 0.039 | 0.36 | 1.0e-05 | 511.5 | 0.051 |
| DIVALIKE | -254.4 | 2 | 0.035 | 0.31 | 0 | 512.9 | 0.025 |
| DIVALIKE+J | -254.5 | 3 | 0.038 | 0.36 | 1.0e-05 | 515.2 | 0.0078 |

Note: The best model was selected with the highest AICc_wt value.

**Table S7.** Characters and character states used in this study.

| Character codes | 1 | 2 | 3 | 4 | 5 | 6 | 7 | 8 | 9 | 10 | 11 | 12 | 13 | 14 |
| --- | --- | --- | --- | --- | --- | --- | --- | --- | --- | --- | --- | --- | --- | --- |
| *Alnus alnobetula* subsp. *alnobetula MF136498* | 0 | 1 | 0 | 0 | 0 | 0 | 0 | 1 | 1 | 0 | 0 | 0 | 0 | 0 |
| *Alnus alnobetula* subsp. *crispa MF136495* | 0 | 1 | 0 | 0 | 0 | 0 | 0 | 1 | 1 | 0 | 0 | 0 | 0 | 0 |
| *Alnus alnobetula* subsp. *maximowiczii MF136512* | 0 | 1 | 0 | 0 | 0 | 0 | 0 | 1 | 1 | 0 | 0 | 0 | 0 | 0 |
| *Alnus alnobetula* subsp. *suaveolens MF136496* | 0 | 1 | 0 | 0 | 0 | 0 | 0 | 1 | 1 | 0 | 0 | 0 | 0 | 0 |
| *Alnus cordata MF136500* | 0 | 1 | 0 | 0 | 0 | 0 | 0 | 1 | 1 | 0 | 0 | 0 | 0 | 0 |
| *Alnus cremastogyne MH628453* | 0 | 1 | 0 | 0 | 0 | 0 | 0 | 1 | 1 | 0 | 0 | 0 | 0 | 0 |
| *Alnus glutinosa MG386363* | 0 | 1 | 0 | 0 | 0 | 0 | 0 | 1 | 1 | 0 | 0 | 0 | 0 | 0 |
| *Alnus glutinosa* subsp. *barbata MF136501* | 0 | 1 | 0 | 0 | 0 | 0 | 0 | 1 | 1 | 0 | 0 | 0 | 0 | 0 |
| *Alnus glutinosa* subsp. *betuloides MF136499* | 0 | 1 | 0 | 0 | 0 | 0 | 0 | 1 | 1 | 0 | 0 | 0 | 0 | 0 |
| *Alnus glutinosa* subsp. *glutinosa MF136504* | 0 | 1 | 0 | 0 | 0 | 0 | 0 | 1 | 1 | 0 | 0 | 0 | 0 | 0 |
| *Alnus incana MF136506* | 0 | 1 | 0 | 0 | 0 | 0 | 0 | 1 | 1 | 0 | 0 | 0 | 0 | 0 |
| *Alnus japonica MF136507* | 0 | 1 | 0 | 0 | 0 | 0 | 0 | 1 | 1 | 0 | 0 | 0 | 0 | 0 |
| *Alnus jorullensis* subsp*. jorullensis MF136508* | 0 | 1 | 0 | 0 | 0 | 0 | 0 | 1 | 1 | 0 | 0 | 0 | 0 | 0 |
| *Alnus maritima* subsp. *maritima MF136510* | 0 | 1 | 0 | 0 | 0 | 0 | 0 | 1 | 1 | 0 | 0 | 0 | 0 | 0 |
| *Alnus maritima* subsp. *oklahomensis MF136511* | 0 | 1 | 0 | 0 | 0 | 0 | 0 | 1 | 1 | 0 | 0 | 0 | 0 | 0 |
| *Alnus nepalensis MG386365* | 0 | 1 | 0 | 0 | 0 | 0 | 0 | 1 | 1 | 0 | 0 | 0 | 0 | 0 |
| *Alnus nitida MF136513* | 0 | 1 | 0 | 0 | 0 | 0 | 0 | 1 | 1 | 0 | 0 | 0 | 0 | 0 |
| *Alnus orientalis MF136514* | 0 | 1 | 0 | 0 | 0 | 0 | 0 | 1 | 1 | 0 | 0 | 0 | 0 | 0 |
| *Alnus rubra MG356709* | 0 | 1 | 0 | 0 | 0 | 0 | 0 | 1 | 1 | 0 | 0 | 0 | 0 | 0 |
| *Alnus subcordata MF136516* | 0 | 1 | 0 | 0 | 0 | 0 | 0 | 1 | 1 | 0 | 0 | 0 | 0 | 0 |
| *Betula alnoides MK888853* | 0 | 1 | 0 | 1 | 1 | 0 | 0 | 1 | 1 | 0 | 1 | 0 | 0 | 0 |
| *Betula chichibuensis LC542973* | 0 | 1 | 0 | 1 | 1 | 0 | 0 | 1 | 1 | 0 | 1 | 0 | 0 | 0 |
| *Betula cordifolia MG386401* | 0 | 1 | 0 | 1 | 1 | 0 | 0 | 1 | 1 | 0 | 1 | 0 | 0 | 0 |
| *Betula costata MN830400* | 0 | 1 | 0 | 1 | 1 | 0 | 0 | 1 | 1 | 0 | 1 | 0 | 0 | 0 |
| *Betula halophila MG674393* | 0 | 1 | 0 | 1 | 1 | 0 | 0 | 1 | 1 | 0 | 1 | 0 | 0 | 0 |
| *Betula lenta MG386366* | 0 | 1 | 0 | 1 | 1 | 0 | 0 | 1 | 1 | 0 | 1 | 0 | 0 | 0 |
| *Betula microphylla MT310900* | 0 | 1 | 0 | 1 | 1 | 0 | 0 | 1 | 1 | 0 | 1 | 0 | 0 | 0 |
| *Betula nana KX703002* | 0 | 1 | 0 | 1 | 1 | 0 | 0 | 1 | 1 | 0 | 1 | 0 | 0 | 0 |
| *Betula occidentalis MG386367* | 0 | 1 | 0 | 1 | 1 | 0 | 0 | 1 | 1 | 0 | 1 | 0 | 0 | 0 |
| *Betula pendula LT996895 1* | 0 | 1 | 0 | 1 | 1 | 0 | 0 | 1 | 1 | 0 | 1 | 0 | 0 | 0 |
| *Betula platyphylla MG386368* | 0 | 1 | 0 | 1 | 1 | 0 | 0 | 1 | 1 | 0 | 1 | 0 | 0 | 0 |
| *Betula populifolia MG386369* | 0 | 1 | 0 | 1 | 1 | 0 | 0 | 1 | 1 | 0 | 1 | 0 | 0 | 0 |
| *Betula pubescens MG386370* | 0 | 1 | 0 | 1 | 1 | 0 | 0 | 1 | 1 | 0 | 1 | 0 | 0 | 0 |
| *Carpinus betulus MF977767* | 1 | 1 | 1 | 1 | 1 | 1 | 1 | 0 | 0 | 1 | 1 | 5 | 0 | 0 |
| *Carpinus caroliniana MF977768* | 1 | 1 | 1 | 1 | 1 | 1 | 1 | 0 | 0 | 1 | 1 | 5 | 0 | 0 |
| *Carpinus cordata MF977769* | 1 | 1 | 1 | 1 | 1 | 1 | 1 | 0 | 0 | 1 | 1 | 5 | 0 | 0 |
| *Carpinus fangiana MF977770* | 1 | 1 | 1 | 1 | 1 | 1 | 1 | 0 | 0 | 1 | 1 | 5 | 0 | 0 |
| *Carpinus fargesiana MF977771* | 1 | 1 | 1 | 1 | 1 | 1 | 1 | 0 | 0 | 1 | 1 | 5 | 0 | 0 |
| *Carpinus hebestroma MG720819* | 1 | 1 | 1 | 1 | 1 | 1 | 1 | 0 | 0 | 1 | 1 | 5 | 0 | 0 |
| *Carpinus laxiflora MK425701* | 1 | 1 | 1 | 1 | 1 | 1 | 1 | 0 | 0 | 1 | 1 | 5 | 0 | 0 |
| *Carpinus monbeigiana MG386372* | 1 | 1 | 1 | 1 | 1 | 1 | 1 | 0 | 0 | 1 | 1 | 5 | 0 | 0 |
| *Carpinus oblongifolia MG720817* | 1 | 1 | 1 | 1 | 1 | 1 | 1 | 0 | 0 | 1 | 1 | 5 | 0 | 0 |
| *Carpinus polyneura MG386373* | 1 | 1 | 1 | 1 | 1 | 1 | 1 | 0 | 0 | 1 | 1 | 5 | 0 | 0 |
| *Carpinus purpurinervis MG720818* | 1 | 1 | 1 | 1 | 1 | 1 | 1 | 0 | 0 | 1 | 1 | 5 | 0 | 0 |
| *Carpinus putoensis KX695124* | 1 | 1 | 1 | 1 | 1 | 1 | 1 | 0 | 0 | 1 | 1 | 5 | 0 | 0 |
| *Carpinus rupestris MG386374* | 1 | 1 | 1 | 1 | 1 | 1 | 1 | 0 | 0 | 1 | 1 | 5 | 0 | 0 |
| *Carpinus tibetana MT727002* | 1 | 1 | 1 | 1 | 1 | 1 | 1 | 0 | 0 | 1 | 1 | 5 | 0 | 0 |
| *Carpinus tientaiensis KY174338 1* | 1 | 1 | 1 | 1 | 1 | 1 | 1 | 0 | 0 | 1 | 1 | 5 | 0 | 0 |
| *Carpinus tschonoskii MF977772* | 1 | 1 | 1 | 1 | 1 | 1 | 1 | 0 | 0 | 1 | 1 | 5 | 0 | 0 |
| *Carpinus viminea MF977773* | 1 | 1 | 1 | 1 | 1 | 1 | 1 | 0 | 0 | 1 | 1 | 5 | 0 | 0 |
| *Corylus americana MH628446* | 0 | 0 | 0 | 0 | 2 | 0 | 1 | 1 | 1 | 1 | 2 | 1 | 1 | 1 |
| *Corylus avellana KX822768 2* | 0 | 0 | 0 | 0 | 2 | 0 | 1 | 1 | 1 | 1 | 2 | 1 | 1 | 1 |
| *Corylus chinensis KX814336 2* | 0 | 0 | 0 | 0 | 2 | 0 | 1 | 1 | 1 | 1 | 2 | 2 | 1 | 1 |
| *Corylus colurna MH628457* | 0 | 0 | 0 | 0 | 2 | 0 | 1 | 1 | 1 | 1 | 2 | 1 | 1 | 1 |
| *Corylus cornuta MH628460* | 0 | 0 | 0 | 0 | 2 | 0 | 1 | 1 | 1 | 1 | 2 | 2 | 1 | 1 |
| *Corylus cornuta* var. *californica MH628459* | 0 | 0 | 0 | 0 | 2 | 0 | 1 | 1 | 1 | 1 | 2 | 2 | 1 | 1 |
| *Corylus fargesii MH628462* | 0 | 0 | 0 | 0 | 2 | 0 | 1 | 1 | 1 | 1 | 2 | 2 | 1 | 1 |
| *Corylus ferox MH628455* | 0 | 0 | 0 | 0 | 2 | 0 | 1 | 1 | 1 | 1 | 2 | 3 | 1 | 1 |
| *Corylus ferox* var. *thibetica MH628450* | 0 | 0 | 0 | 0 | 2 | 0 | 1 | 1 | 1 | 1 | 2 | 3 | 1 | 1 |
| *Corylus heterophylla KX822769 2* | 0 | 0 | 0 | 0 | 2 | 0 | 1 | 1 | 1 | 1 | 2 | 1 | 1 | 1 |
| *Corylus heterophylla* var. *sutchuenensis MH628447* | 0 | 0 | 0 | 0 | 2 | 0 | 1 | 1 | 1 | 1 | 2 | 1 | 1 | 1 |
| *Corylus jacquemontii MH628458* | 0 | 0 | 0 | 0 | 2 | 0 | 1 | 1 | 1 | 1 | 2 | 1 | 1 | 1 |
| *Corylus kweichowensis* var. *brevipes MH628452* | 0 | 0 | 0 | 0 | 2 | 0 | 1 | 1 | 1 | 1 | 2 | 1 | 1 | 1 |
| *Corylus mandshurica MH628448* | 0 | 0 | 0 | 0 | 2 | 0 | 1 | 1 | 1 | 1 | 2 | 2 | 1 | 1 |
| *Corylus sieboldiana MH628456* | 0 | 0 | 0 | 0 | 2 | 0 | 1 | 1 | 1 | 1 | 2 | 2 | 1 | 1 |
| *Corylus wangii MH628454* | 0 | 0 | 0 | 0 | 2 | 0 | 1 | 1 | 1 | 1 | 2 | 1 | 1 | 1 |
| *Corylus yunnanensis MF375336* | 0 | 0 | 0 | 0 | 2 | 0 | 1 | 1 | 1 | 1 | 2 | 1 | 1 | 1 |
| *Ostrya chinensis MG662156* | 1 | 1 | 1 | 0 | 1 | 1 | 1 | 0 | 1 | 1 | 2 | 4 | 0 | 0 |
| *Ostrya japonica MG662142* | 1 | 1 | 1 | 0 | 1 | 1 | 1 | 0 | 1 | 1 | 2 | 4 | 0 | 0 |
| *Ostrya rehderiana MG662133* | 1 | 1 | 1 | 0 | 1 | 1 | 1 | 0 | 1 | 1 | 2 | 4 | 0 | 0 |
| *Ostrya trichocarpa KY088271* | 1 | 1 | 1 | 0 | 1 | 1 | 1 | 0 | 1 | 1 | 2 | 4 | 0 | 0 |
| *Ostryopsis davidiana MH628451* | 1 | 0 | 1 | 0 | 1 | 0 | 1 | 1 | 1 | 0 | 2 | 4 | 0 | 0 |
| *Ostryopsis intermedia MG386377* | 1 | 0 | 1 | 0 | 1 | 0 | 1 | 1 | 1 | 0 | 2 | 4 | 0 | 0 |
| *Ostryopsis nobilis MG386378* | 1 | 0 | 1 | 0 | 1 | 0 | 1 | 1 | 1 | 0 | 2 | 4 | 0 | 0 |

1. Inflorescence: 0 = bisexual, 1 = unisexual.
2. Infructescence types: 0 = aggregated cluster, 1 = raceme.
3. Staminate perianth: 0 = present, 1 = absent.
4. Pistilloide in male floret: 0 = present, 1 = absent.
5. Thecae and filaments: 0 = thecae and filaments not divided longitudinally, 1 = thecae separated, filaments partly divided, 2 = thecae separated, filaments completely divided.
6. Vessel perforations: 0 = scalariform, 1 = simple.
7. Tracheids: 0 = present, 1 = absent.
8. Tyloses: 0 = present, 1 = absent.
9. Stomatal apparatus: 0 = present, 1 = absent.
10. Embedded glands on leaves: 0 = present, 1 = absent.
11. Leaf teeth: 0 = absent, 1 = simple, 2 = compound.
12. Shape of fruit bracts: 0 = winglike, 1 = campanulate, 2 = tubular, 3 = spiny, 4 = saclike, 5 = leafy.
13. Diaspore types: 0 = winged, 1 = wingless.
14. Seed germination: 0 = epigeal, 1 = hypogeal.
